# Clinical utility of the Fibrosis-4 index for predicting mortality in patients with heart failure with or without metabolic dysfunction-associated steatotic liver disease: a prospective cohort study

**DOI:** 10.1016/j.lanepe.2024.101153

**Published:** 2024-11-30

**Authors:** Joost Boeckmans, Jürgen H. Prochaska, Alexander Gieswinkel, Michael Böhm, Philipp S. Wild, Jörn M. Schattenberg

**Affiliations:** a Metabolic Liver Research Center, I. Department of Medicine, University Medical Center Mainz, Mainz, Germany; b In Vitro Liver Disease Modelling Team, Department of In Vitro Toxicology and Dermato-Cosmetology, Faculty of Medicine and Pharmacy, Vrije Universiteit Brussel, Brussels, Belgium; c Preventive Cardiology and Preventive Medicine, Department of Cardiology, University Medical Center of the Johannes Gutenberg University Mainz, Mainz, Germany; d German Center for Cardiovascular Research (DZHK), Partnersite Rhine-Main, University Medical Center Mainz, Johannes Gutenberg University Mainz, Germany; e Center for Thrombosis and Hemostasis, University Medical Center of the Johannes Gutenberg University Mainz, Germany; f Department of Medicine III, University Medical Center Homburg, Homburg and Saarland University, Saarbrücken, Germany; g Systems Medicine, Institute of Molecular Biology (IMB), Mainz, Germany; h Department of Medicine II, University Medical Center Homburg, Homburg and Saarland University, Saarbrücken, Germany

**Keywords:** Non-invasive test, Liver fibrosis, Cardiovascular disease, Multidisciplinary care

## Abstract

**Background:**

The liver–heart axis potentially influences the risk of mortality in patients with heart failure. We aimed to identify the clinical utility of the fibrosis-4 (FIB-4) index in patients with heart failure for predicting mortality in the context of metabolic dysfunction-associated steatotic liver disease (MASLD).

**Methods:**

Patients with heart failure and a subsample of healthy participants were enrolled in the MyoVasc study (NCT04064450) and followed for nine years. Participants with excessive alcohol consumption were excluded. The Fatty Liver Index (FLI) and FIB-4 index were used to classify MASLD and hepatic fibrosis, respectively. Data were adjusted for potential confounders. The primary endpoint was all-cause mortality.

**Findings:**

2726 participants, including 172 healthy individuals, were included in the study. The participants had a mean age of 64.4 ± 11.2 years and a median FIB-4 index of 1.59 (interquartile range [1.17; 2.17]). There were 532 deaths. The FIB-4 index was predictive for all-cause mortality (hazard ratio (HR) 1.341, 95% confidence interval (CI) [1.273; 1.412], p < 0.0001). The HRs and 95% CIs for the FIB-4 index in FLI categories were 1.597 [1.256; 2.031] (p = 0.00013, FLI <30), 1.802 [1.519; 2.138] (p < 0.0001, FLI 30–60), and 1.292 [1.215; 1.374] (p < 0.0001, FLI ≥60). The interaction term for the FIB-4 index with FLI ≥60 (reference FLI <30) was HR 0.774 [0.617; 0.972] (p = 0.027), indicating a smaller impact of the FIB-4 index in FLI ≥60 than in FLI <30 (HR 1.664 [1.333; 2.077], p < 0.0001). Multivariable linear regressions revealed relevant independent relationships between the FIB-4 index and N-terminal pro-B-type natriuretic peptide, systolic dysfunction, diastolic dysfunction and left ventricular hypertrophy in participants with a FLI below 60.

**Interpretation:**

In patients with heart failure, the FIB-4 index predicts all-cause mortality and relates to cardiac functional and structural changes, especially in those without MASLD.

**Funding:**

Johannes Gutenberg-University Mainz.

**Research in context**

**Evidence before this study**

PubMed was searched for articles published between 1 June 2006 (when the Fibrosis-4 (FIB-4) index was first published) and 31 March 2024 that describe to prognostic value of the FIB-4 index for predicting mortality in patients with heart failure in the context of metabolic dysfunction-associated steatotic liver disease (MASLD). Search terms were (“FIB-4” OR “Fibrosis-4” OR “Fibrosis-4 index”) AND (“heart failure”) AND (“mortality”) without language restriction. Although no studies were found that describe the use of the FIB-4 index in patients with heart failure with underlying MASLD, several studies were retrieved that describe the potential clinical utility of the FIB-4 index for predicting mortality in patients heart failure. Most studies were observational cohort studies and conducted in Asia. Importantly, the FIB-4 index was predictive for mortality in patients that were discharged from the hospital after cardiac decompensation using the FIB-4 index as a continuous variable and as tertiles. Since the FIB-4 index is a readily accessible and inexpensive marker in both primary and specialty care, it would be a convenient tool to predict cardiac and hepatic reserve in daily clinical practice in the context of multidisciplinary disease management.

**Added value of this study**

Using one of the largest prospective cohorts of patients with heart failure in Europe with thorough cardiac characterization using echocardiography and follow-up of nine years, we confirmed the prognostic utility of the FIB-4 index for predicting mortality in patients with heart failure. Further, the FIB-4 index was related to N-terminal pro-B-type natriuretic peptide levels, left ventricular hypertrophy, systolic dysfunction, and diastolic dysfunction. The relationships between the FIB-4 index and cardiac parameters and mortality were the strongest in the absence of MASLD. In addition, data were adjusted for common metabolic and cardiovascular diseases, indicating that the FIB-4 index is an independent predictor of cardiac structural and functional changes and mortality, and that its use in clinical practice can aid the multidisciplinary management of metabolic liver and cardiovascular disease.

**Implications of all the available evidence**

The FIB-4 index predicts mortality and cardiac impairment in patients with heart failure and can hence be a convenient tool in clinical practice for guiding the multidisciplinary management of patients with heart failure, also in less-resourced settings.

## Introduction

Metabolic dysfunction-associated steatotic liver disease (MASLD) is an umbrella term ranging from isolated liver steatosis to progressive metabolic dysfunction-associated steatohepatitis (MASH), liver fibrosis, and cirrhosis in the absence of excessive alcohol consumption or other causes of fatty liver.^1^ In MASLD, cardiovascular-related death occurs often before liver-related death can occur and is hence a leading cause of mortality in these patients.^2^^,^^3^ MASLD increases the risk to develop heart failure (HF) by approximately 1.5 times and is therefore closely linked with it, irrespective of other cardiovascular risk factors.^4^ Furthermore, a pooled analysis showed that MASLD increases the risk of all-cause mortality in patients with HF (hazard ratio (HR): 1.66, 95% confidence interval (CI) [1.39; 1.98]),^5^ suggesting that it is an independent risk factor.

MASLD and HF are highly prevalent in the general population (MASLD 38%^6^ and HF 1–2%^7^) and can both lead to fibrogenesis and stiffening of the liver in advanced disease, although based on different pathophysiological mechanisms.^1^^,^^8^ Screening for advanced hepatic fibrosis in metabolically-compromised patients using non-invasive tests (NITs), as recommended by current guidelines, is hence complicated when accompanied by HF.^9^^,^^10^ The fibrosis-4 (FIB-4) index is the recommended first-line screening test and has been explored for its predictive ability in HF. In a cohort of 1058 hospital-admitted patients with decompensated chronic HF and a mean follow-up of 1047 days, the FIB-4 index as a continuous measure and as categories (<1.72, ≥1.72 and < 3.01 and 3.01 ≤) predicted all-cause mortality,^11^ suggesting that hepatic fibrosis could add to the mortality risk in chronic HF. The primary origin of liver fibrosis in HF that drives mortality remains however elusive. Hepatic-derived, metabolic inflammation could be the underlying pathophysiological mechanism connecting the liver and heart to cardiac remodeling.^3^^,^^12^

The accuracy of a NIT is influenced by the prevalence of the condition in the study cohort. As a result, the prognostic value of the FIB-4 index in patients with chronic HF and MASLD remains unknown. As MASLD is a slowly progressing disease,^13^ and hepatic congestion can result in liver fibrosis,^8^ we hypothesized that the FIB-4 index especially predicts mortality and cardiac impairment in those without MASLD.

This large prospective cohort study aimed to evaluate the FIB-4 index in its ability to predict all-cause mortality in patients with chronic HF and to position this in the context of MASLD.

## Methods

### Study protocol and sample

Results were obtained from the MyoVasc study, a single-center prospective cohort study based in the University Medical Center of the Johannes Gutenberg-University Mainz (Germany). The study was prospectively registered on ClinicalTrials.gov with registration number NCT04064450, and J.P. and P.S.W. were involved in its initial design. This study is a secondary analysis of the MyoVasc study. All study participants gave their written informed consent before enrolling in the study. The study was conducted according to the Declaration of Helsinki, Good Clinical Practice, and Good Epidemiological Practice. Ethical and study approvals were obtained from the responsible ethics committee with reference number 837.319.12 (8420-F) and the data safety commissioner. The study is reported according to the STROBE guidelines (checklist in Supplementary Material).

Patients covering the full spectrum of chronic HF and a subsample of participants without cardiovascular risk factors were enrolled between January 2013 and April 2018. Participants were mostly of white ancestry and derived from a mid-Western European population. Of all study participants (n = 3289), there were 295 individuals (9.0%) free from cardiovascular risk factors. For the present study, the subsample of participants free from cardiovascular risk factors was retained in the study sample to have extreme phenotypes at both ends of the HF spectrum and generate more precise estimates. Details on follow-up assessments have been extensively reported elsewhere.^14^ Participants daily consuming significant amounts of alcohol were excluded, being more than 30 g for men, and 20 g for women.^9^^,^^10^

### Data sources

Transthoracic resting two-dimensional echocardiograms were made using an iE33 echocardiography system (Royal Philips Electronics, Amsterdam (The Netherlands)) in line with the recommendations of the American and European societies of echocardiography.^15^ Left ventricular ejection fraction (LVEF), lateral E/E′-ratio, relative wall thickness (RWT), and left ventricular mass indexed to height^2.7^ (LVMI) were acquired using an integrated multi-modality image management system for cardiovascular information (Xcelera, Philips Healthcare, Hamburg, Germany).

Venous blood for determination of liver- and heart-related blood parameters was analyzed in the central laboratory of the Institute for Clinical Chemistry and Laboratory Medicine, University Medical Center Mainz (Germany).

All investigations were done on the same day for each individual participant in the study center.

### Definitions of diseases

The FIB-4 index was calculated at baseline according to Sterling et al. and the fatty liver index (FLI) was calculated at baseline according to Bedogni et al.^16^^,^^17^ For the purpose of this study, a FIB-4 index below 1.3 was considered as ‘no advanced hepatic fibrosis’ while a FIB-4 index of at least 2.67 was considered as ‘advanced hepatic fibrosis’.^18^ An FLI below 30 was considered as ‘no MASLD’ while an FLI of at least 60 was considered as ‘evidence for MASLD’.^17^ ‘HF’ and HF phenotypes were defined according to the universal definition and classification of HF.^19^ ‘HF’ was considered as stage B, C, or D while ‘no HF’ was defined as HF stage 0 (*i.e.* no HF but not reaching the criteria of stage A) or A. Definitions of risk factors, comorbidities, and medication are provided in the Supplementary Material.

### Definitions of outcomes

The primary outcome of this investigation was all-cause mortality. Information on death was obtained via quarterly checks with the German death register, which is continuously updated.

### Statistical analyses

Continuous Gaussian-distributed data were presented as mean ± standard deviation (SD) and continuous non-Gaussian distributed data were presented as median ± interquartile range (IQR). Discrete data were described as absolute and relative frequencies.

A chi-square was used to compare trends in categorical data. For all cross-sectional and longitudinal analyses, the FIB-4 index (or one of its constituents) was the independent variable. The associations between the FIB-4 index and N-terminal pro-B-type natriuretic peptide (NT-proBNP), LVEF, lateral E/E’ ratio, LVMI, and RWT were determined using multivariable linear regression analyses for which β-estimates with 95% confidence intervals were reported. β-estimates were reported per 1 SD. All-cause mortality was analyzed over time using Kaplan–Meier analysis with a log-rank test to test differences between FIB-4 index categories. Multivariable Cox regression analyses were used to generate HRs with 95% CIs using the FIB-4 index as a continuous and categorical variable using cutoffs of 1.3, 2.0, and 2.67. The effect of the FIB-4 index on all-cause mortality according to FLI categories (FLI <30, FLI 30–60, and FLI ≥60) was explored using the FIB-4 index on a continuous scale. To provide granularity on the data, Cox regressions were also done for the FIB-4 constituents. Participants were censored when they were lost to follow-up. Follow-up ended at death or upon reaching the end of the study period.

Two models (nested) were employed for the cross-sectional analyses. Model 1 had adjustment for age (continuous) and sex (binary); model 2 had additional adjustment for arterial hypertension, diabetes mellitus, smoking, obesity, dyslipidemia, family history of myocardial infarction/stroke (all binary), and C-reactive protein (CRP) (continuous). For the Cox regression analyses, three models (nested) were used. Models 1 and 2 were the same as in the cross-sectional analyses, but without CRP; model 3 had additional adjustment for cancer, atrial fibrillation, chronic kidney disease, coronary artery disease, myocardial infarction, peripheral artery disease, and stroke (all binary). Data were described in the text in model 2 unless otherwise indicated.

Sensitivity analyses were performed for heart failure phenotypes (age- and sex-adjusted). Additionally, interaction terms for the FIB-4 index (continuous) between the FLI categories (FLI <30 as reference) were calculated for the primary outcome of all-cause mortality. p-values lower than 0.05 were considered statistically significant. Missing data were not imputed. Missing data at baseline is reported in Supplementary Table S1. Assumptions on the Cox proportional hazards models and multivariable linear regression models were checked and no violations were found (Supplementary Figures S1–S6). Statistical analyses were performed using the R software package (https://www.R-project.org, Version 4.2.1).

### Role of funding source

The funders of the study had no role in the study design, data collection, data analysis, data interpretation, writing of the report, or in the decision to submit the paper for publication.

## Results

### Characteristics of the study participants

The MyoVasc study cohort enrolled 3289 participants between January 2013 and April 2018. For the analysis of MASLD, we excluded 513 individuals who consumed more than 30 g of alcohol for men and 20 g for women, and 50 individuals due to lack of FIB-4 data, resulting in a sample of 2726 subjects for the analysis (HF stage C/D n = 1,488, HF stage B n = 767, HF stage A n = 299, healthy participants n = 172). The baseline clinical and laboratory characteristics of the study participants are shown in Table 1. The study participants had a mean age of 64.4 ± 11.2 years and included 37.9% women. Symptomatic heart failure was present in 58.8% of the study participants. HF with preserved ejection fraction (HFpEF) was the most prevalent HF phenotype in the study sample, covering 624 study participants. The median FIB-4 index was 1.59 (IQR [1.17; 2.17]) and the mean FLI was 56.18 ± 29.51.

**Table 1:** Baseline clinical and laboratory characteristics according to the FIB-4 index.

Variable	All (2726)	FIB-4 < 1.3 (894)	FIB-4 1.3–2.67 (1468)	FIB-4 ≥ 2.67 (364)
Sex (Women)	37.9% (n: 1033/2726)	44.9% (n: 401/894)	36.9% (n: 542/1468)	24.7% (n: 90/364)
Age [year]	64.4 (11.2)	55.5 (10.4)	67.6 (8.7)	73.2 (7.7)
Body mass index [kg/m^2^]	28.3 (5.0)	28.5 (5.4)	28.4 (4.9)	27.8 (4.5)
Systolic blood pressure [mmHg]	131.5 (18.0)	128.6 (16.8)	132.6 (17.9)	134.3 (20.2)
Diastolic blood pressure [mmHg]	77.6 (10.0)	79.0 (9.9)	77.0 (9.7)	76.2 (11.1)
Heart rate [bpm]	63.1 (10.7)	64.3 (9.7)	62.3 (10.8)	63.0 (12.0)
**FLI and FIB-4**				
FLI	56.18 (29.51)	54.99 (31.08)	56.32 (28.84)	58.53 (28.11)
FIB-4	1.59 (1.17/2.17)	1.04 (0.86/1.17)	1.78 (1.53/2.12)	3.17 (2.89/3.64)
**Cardiovascular risk factors**				
Arterial hypertension	71.5% (n: 1948/2726)	59.5% (n: 532/894)	76.3% (n: 1120/1468)	81.3% (n: 296/364)
Diabetes mellitus	23.2% (n: 632/2726)	17.7% (n: 158/894)	24.6% (n: 361/1468)	31.0% (n: 113/364)
Smoking	12.6% (n: 344/2726)	18.5% (n: 165/894)	9.7% (n: 143/1468)	9.9% (n: 36/364)
Obesity	31.9% (n: 870/2726)	33.1% (n: 296/894)	32.2% (n: 472/1468)	28.0% (n: 102/364)
Dyslipidemia	69.0% (n: 1882/2726)	63.2% (n: 565/894)	71.3% (n: 1047/1468)	74.2% (n: 270/364)
Family history of myocardial infarction/stroke	23.3% (n: 636/2725)	26.2% (n: 234/893)	21.7% (n: 318/1468)	23.1% (n: 84/364)
**Comorbidities**				
History of myocardial infarction	24.6% (n: 670/2726)	20.1% (n: 180/894)	25.6% (n: 376/1468)	31.3% (n: 114/364)
History of stroke	8.7% (n: 236/2726)	6.8% (n: 61/894)	9.1% (n: 133/1468)	11.5% (n: 42/364)
Coronary artery disease	38.6% (n: 1052/2726)	28.4% (n: 254/894)	41.3% (n: 607/1468)	52.5% (n: 191/364)
Atrial fibrillation	23.4% (n: 638/2726)	11.7% (n: 105/894)	26.2% (n: 385/1468)	40.7% (n: 148/364)
Peripheral artery disease	6.6% (n: 179/2726)	4.9% (n: 44/894)	6.5% (n: 95/1468)	11.0% (n: 40/364)
Chronic kidney disease	17.9% (n: 487/2726)	11.9% (n: 106/894)	18.4% (n: 270/1468)	30.5% (n: 111/364)
Cancer	16.4% (n: 448/2726)	10.3% (n: 92/894)	18.5% (n: 271/1468)	23.4% (n: 85/364)
**Laboratory parameters**				
Cholesterol [mmol/l]	200.6 (47.2)	210.1 (46.1)	200.0 (46.7)	179.8 (44.7)
High-density lipoprotein [mg/dl]	52.6 (15.3)	53.3 (15.4)	52.9 (15.3)	49.7 (14.8)
Low-density lipoprotein [mg/dl]	122.1 (40.0)	130.2 (39.6)	121.2 (39.7)	105.8 (36.4)
Triglycerides [mg/dl]	111.0 (80.0/157.0)	117.0 (80.0/166.0)	111.0 (81.0/156.0)	102.0 (79.0/141.6)
eGFR [ml/min/1.73 m^2^]	77.63 (20.02)	86.63 (18.26)	74.93 (18.71)	66.41 (20.49)
Aspartate aminotransferase [U/l]	26.0 (23.0/31.0)	24.0 (21.0/29.0)	26.0 (23.0/31.0)	32.0 (27.0/38.0)
Alanine aminotransferase [U/l]	23.00 (17.00/31.00)	25.00 (18.00/35.00)	22.00 (17.00/29.00)	22.00 (16.00/30.58)
HbA1c [%]	5.70 (5.40/6.20)	5.60 (5.40/6.00)	5.80 (5.50/6.20)	5.90 (5.50/6.40)
N-terminal pro–B-type natriuretic peptide [pg/ml]	169.00 (70.00/478.25)	87.00 (45.00/204.87)	197.00 (85.00/519.33)	534.50 (202.83/1493.67)
C-reactive protein [mg/L]	1.80 (0.89/3.80)	1.80 (0.89/3.70)	1.70 (0.88/3.70)	2.00 (0.95/4.70)
**Cardiac function and structure**				
Left ventricular ejection fraction [%]	54.4 (11.1)	55.8 (10.2)	54.5 (11.1)	50.2 (12.4)
Left ventricular lateral E/E′ ratio	8.44 (6.41/11.30)	7.31 (5.71/9.55)	8.71 (6.71/11.50)	10.15 (7.88/13.84)
Left ventricular mass [g]	189.9 (150.6/242.5)	182.1 (146.0/235.4)	190.0 (151.6/240.7)	214.8 (168.0/260.3)
Left ventricular mass/height [g/m^2.7^]	44.7 (36.6/55.3)	42.2 (35.1/53.5)	44.9 (36.8/55.3)	50.3 (40.5/60.5)
Relative wall thickness	0.42 (0.12)	0.42 (0.12)	0.42 (0.12)	0.43 (0.14)
**Symptomatic heart failure**				
Symptomatic heart failure (stage C or D)	58.8% (n: 1488/2532)	50.3% (n: 380/756)	58.4% (n: 824/1412)	78.0% (n: 284/364)
**Heart failure phenotype**				
Heart failure with preserved ejection fraction	22.9% (624/2726)	15.4% (138/894)	25.5% (375/1468)	30.5% (111/364)
Heart failure with midrange ejection fraction	12.4% (338/2726)	11.4% (102/894)	12.3% (180/1468)	15.4% (56/364)
Heart failure with reduced ejection fraction	11.1% (302/2726)	7.2% (64/894)	10.6% (155/1468)	22.8% (83/364)
**Medication**				
Antidiabetic medication	16.6% (453/2726)	13.6% (122/894)	18.1% (266/1468)	17.9% (65/364)
Lipid-modifying agents	46.4% (1266/2726)	34.2% (306/894)	50.7% (745/1468)	59.1% (215/364)
Agents acting on the RAS	63.3% (1726/2726)	53.4% (477/894)	67.2% (986/1468)	72.3% (263/364)
Beta blockers	54.8% (1495/2726)	43.8% (392/894)	58.3% (856/1468)	67.9% (247/364)
Calcium channel blocker	17.0% (464/2726)	14.0% (125/894)	18.0% (264/1468)	20.6% (75/364)
Digitalis glycosides, anti-arrhythmics, and vasodilators	17.1% (467/2726)	13.4% (120/894)	18.1% (266/1468)	22.3% (81/364)
Antithrombotic agents	62.2% (1695/2726)	46.0% (411/894)	67.2% (986/1468)	81.9% (298/364)

Abbreviations: FIB-4: fibrosis-4; FLI: fatty liver index, RAS, renin-angiotensin system.

Data presented as mean ± standard deviation (Gaussian-distributed data), median with interquartile range (non-Gaussian distributed data), or as relative and absolute frequencies (categorical data).

Participants with advanced hepatic fibrosis (FIB-4 index ≥2.67) accounted for 13% of the study sample, while in 33% there was a low probability of advanced hepatic fibrosis (FIB-4 index <1.3). Individuals with advanced hepatic fibrosis were older (73.2 ± 7.7 years) than individuals without advanced hepatic fibrosis (FIB-4 index <1.3: 55.5 ± 10.4 years; FIB-4 index 1.3–2.67: 67.6 ± 8.7 years). In addition, participants with advanced hepatic fibrosis had a higher burden of comorbidities, including diabetes mellitus (FIB-4 index <1.3: 17.7%, FIB-4 index 1.3–2.67: 24.6% and FIB-4 index ≥2.67: 31.0%), arterial hypertension (FIB-4 index <1.3: 59.5%, FIB-4 index 1.3–2.67: 76.3% and FIB-4 index ≥2.67: 81.3%), and dyslipidemia (FIB-4 index <1.3: 63.2%, FIB-4 index 1.3–2.67: 71.3% and FIB-4 index ≥2.67: 74.2%). Additionally, a history of cancer (FIB-4 index <1.3: 10.3%, FIB-4 index 1.3–2.67: 18.5% and FIB-4 index ≥2.67: 23.4%), was also more prevalent at baseline in participants with advanced hepatic fibrosis. The LVEF was the lowest in individuals with advanced hepatic fibrosis (50.2 ± 12.4%) and they also had the highest lateral E/E’ ratio (10.15 (7.88/13.84)), suggesting systolic and diastolic dysfunction, respectively.

### Distribution of HF in the study population based on the FIB-4 index and the presence of MASLD

The distribution of prevalent HF was explored based on the FIB-4 index and the presence or absence of MASLD (Supplementary Table S2). Participants without advanced hepatic fibrosis consisted of 55.2% of all individuals without HF, while it included 28.1% of all patients with HF. In contrast, participants with advanced hepatic fibrosis suffered proportionally more often from HF (2.5% of all patients without HF *vs* 15.6% of all patients with HF, p < 0.0001).

When stratified by FLI, individuals without advanced hepatic fibrosis or MASLD (FIB-4 index <1.3 and FLI <30) comprised 23.9% of all participants without HF and 5.8% of all patients with HF (p < 0.0001). Participants with advanced hepatic fibrosis but without MASLD more frequently suffered from HF (3.3% of all patients with HF *vs* 0.2% of all individuals without HF). Prevalent HF was comparable in participants with MASLD without advanced hepatic fibrosis (16.4% of all participants without HF *vs* 16.1% of all participants with HF, p = 0.84), but more prevalent in those with advanced hepatic fibrosis (8.2% of all participants with HF *vs* 1.7% of all participants without HF, p < 0.0001).

### Relationship between the FIB-4 index and NT-proBNP levels based on the presence of MASLD

**Fig. 1:**
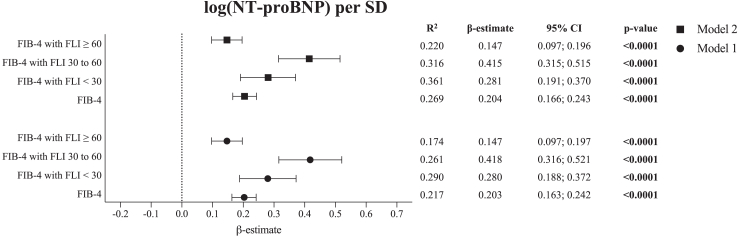
**Relationship between the FIB-4 index and NT-proBNP levels, stratified by FLI categories**. Symbols represent β-estimates and bars represent 95% confidence intervals; p < 0.05 is considered as statistically significant (multivariable linear regression) [abbreviations: CI, confidence interval; FIB-4, fibrosis-4; FLI, fatty liver index; NT-proBNP, N-terminal pro-B-type natriuretic peptide; SD, standard deviation].

Next, the relationship between the FIB-4 index and NT-proBNP levels was investigated in the context of MASLD using multivariable linear regression analysis employing two models (Fig. 1). In the full study sample (β-estimate for the FIB-4 index with log (NT-proBNP) levels 0.204 (95% CI [0.166; 0.243], p < 0.0001) and all FLI categories, the FIB-4 index was a significant predictor for log (NT-proBNP) levels, and not explained by clinical profile or an intermediate function by means of CRP.

The estimated regression coefficients for the FIB-4 index with log (NT-proBNP) levels were approximately two and three times lower in participants with MASLD (β-estimate 0.147 (95% CI [0.097; 0.196], p < 0.0001) compared to participants with an FLI below 30 and 30–60, respectively (FLI <30: β-estimate 0.281 (95% CI [0.191; 0.370], p < 0.0001; FLI 30–60: β-estimate 0.415 (95% CI [0.315; 0.515], p < 0.0001)).

### Relationship between the FIB-4 index and measures of cardiac function based on the presence of MASLD

**Fig. 2:**
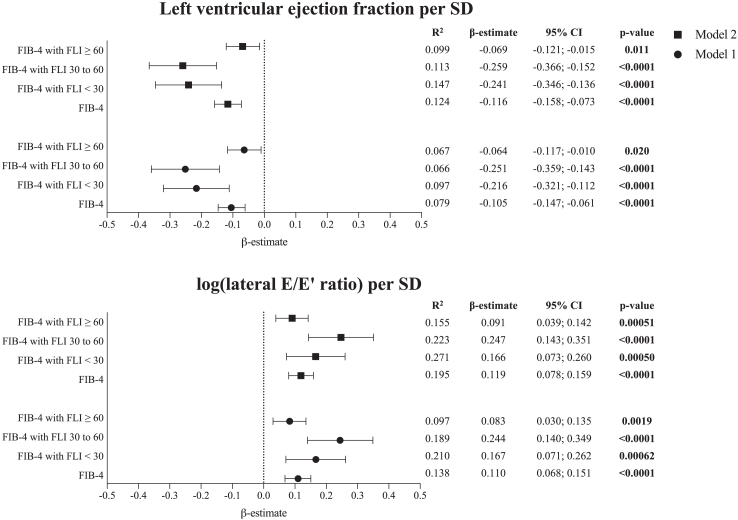
**Relationship between the FIB-4 index and measures of cardiac function, stratified by FLI categories.** Symbols represent β-estimates and bars represent 95% confidence intervals; p < 0.05 is considered as statistically significant (multivariable linear regression). [abbreviations: CI, confidence interval; FIB-4, fibrosis-4; FLI, fatty liver index; SD, standard deviation].

The relationship between the FIB-4 index and systolic function was investigated performing multivariable linear regression analyses with the LVEF. Although the FIB-4 index was associated with LVEF in the whole sample with a β-estimate of −0.116 (95% CI [−0.158; −0.073]), p < 0.0001, differences were observed based on the presence of MASLD (Fig. 2). When stratifying the cohort according to FLI categories, it was observed that the negative association between the FIB-4 index and LVEF was mainly determined by participants having an FLI below 60 (FLI <30: β-estimate −0.241 (95% CI [−0.346; −0.136], p < 0.0001; FLI 30–60: β-estimate −0.259 (95% CI [−0.366; −0.152], p < 0.0001)) and only marginally by participants having MASLD (FLI ≥60: β-estimate −0.069 (95% CI [−0.121; −0.015], p = 0.011).

A similar pattern was observed exploring left ventricular diastolic dysfunction defined by the log (lateral E/E′ ratio) (Fig. 2). While the relationship between the FIB-4 index and log (lateral E/E’ ratio) existed with a β-estimate of 0.119 (95% CI [0.078; 0.159], p < 0.0001) in the full sample, it was largely explained by those having an FLI below 60 (FLI <30: β-estimate 0.166 (95% CI [0.073; 0.260], p = 0.00050; FLI 30–60: β-estimate 0.247 (95% CI [0.143; 0.351] p < 0.0001)) and only to a limited extent by individuals with evidence of MASLD (β-estimate 0.091 (95% CI [0.039; 0.142], p = 0.00051)).

### Relationship between the FIB-4 index and measures of cardiac structure based on the presence MASLD

**Fig. 3:**
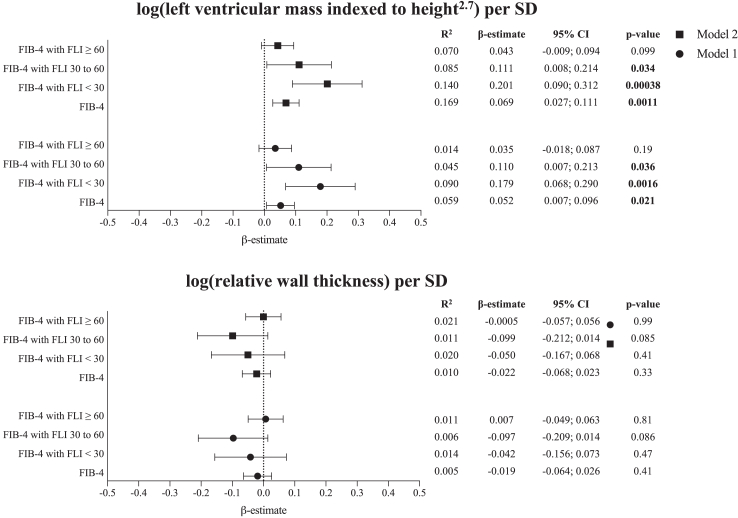
**Relationship between the FIB-4 index and measures of cardiac structure, stratified by FLI categories.** Symbols represent β-estimates and bars represent 95% confidence intervals; p < 0.05 is considered as statistically significant (multivariable linear regression). [abbreviations: CI, confidence interval; FIB-4, fibrosis-4; FLI, fatty liver index; SD, standard deviation].

The relationship between the FIB-4 index and left ventricular hypertrophy was investigated using the LVMI. The FIB-4 index was a significant predictor for log (LVMI) with a beta-estimate of 0.069 (95% CI [0.027; 0.111], p = 0.0011) (Fig. 3). In participants with MASLD, no significant connection between the FIB-4 index and log (LVMI) was found. On the contrary, a β-estimate of 0.201 (95% CI [0.090; 0.312], p = 0.00038) was observed in participants without MASLD, while the FIB-4 index in participants with an FLI from 30 to 60 was associated with log (LVMI) with a β-estimate of 0.111 (95% CI [0.008; 0.214], p = 0.034). No significant associations were found between the FIB-4 index and log (RWT) (Fig. 3).

### Survival analysis of patients with heart failure according to the FIB-4 index based on the presence of MASLD

**Fig. 4:**
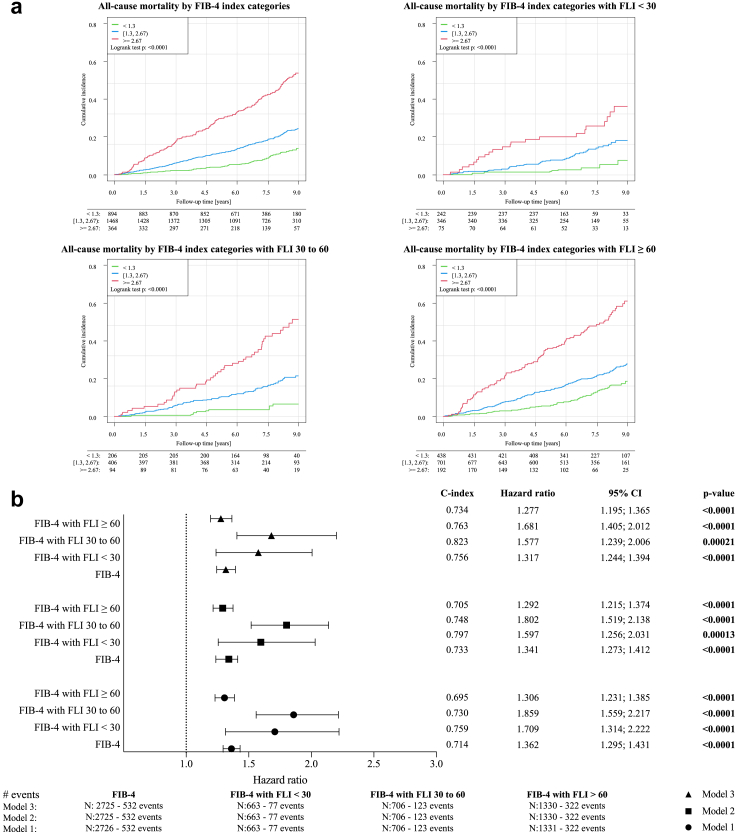
**Longitudinal relationship between the FIB-4 index and all-cause mortality in patients with heart failure in the context of metabolic dysfunction-associated steatotic liver disease** (a) All-cause mortality in patients with heart failure according to FIB-4 index categories and stratified by FLI categories; p < 0.05 is considered as statistically significant (Kaplan–Meier analysis with log rank test) (y-axis: cumulative incidence; x-axis: time in years). (b) Longitudinal analysis for all-cause mortality in patients with heart failure according to the FIB-4 index (continuous) and stratified by FLI categories (symbols represent hazard ratios and bars represent 95% confidence intervals); p < 0.05 is considered as statistically significant (Cox regression). [abbreviations: CI, confidence interval, FIB-4, fibrosis-4; FLI, fatty liver index].

All-cause mortality was investigated in the MyoVasc study cohort with a follow-up period of 108 months. Five hundred thirty-two events occurred. Kaplan–Meier survival analysis showed that the FIB-4 index was predictive for all-cause mortality in HF with and without MASLD (Log-rank tests, p < 0.0001) (Fig. 4a). Cox regression with the FIB-4 index as a continuous variable was used to adjust for possible confounders in three different models (Fig. 4b). The FIB-4 index was an independent predictor for all-cause mortality in all FLI categories (FLI <30 (77 events), FLI 30–60 (123 events) and FLI ≥60 (322 events)) and all models. To avoid overfitting, we used model 2 (adjustment for age, sex, arterial hypertension, diabetes mellitus, smoking, obesity, dyslipidemia and family history of myocardial infarction/stroke) to describe the data. Using the FIB-4 index without stratifying by FLI categories, a HR of 1.341 (95% CI [1.273; 1.412], p < 0.0001) was obtained. Yet, the lowest HR was observed in the highest FLI category (FIB-4 index with FLI <30: HR 1.597 (95% CI [1.256; 2.031], p = 0.00013), FIB-4 index with FLI 30–60: HR 1.802 (95% CI [1.519; 2.138], p < 0.0001), and FIB-4 index with FLI ≥60: HR 1.292 (95% CI [1.215; 1.374], p < 0.0001)). The same trend was observed in model 3 (Fig. 4b).

The interaction term for the FIB-4 index in participants with FLI ≥60 was 0.774 (95% CI [0.617; 0.972], p = 0.027), indicating a smaller impact of the FIB-4 index in participants with FLI ≥60 than in FLI <30 (HR 1.664 (95% CI [1.333; 2.077], p < 0.0001), although still being predictive of all-cause mortality (1.664 × 0.774 = HR 1.288) (Supplementary Table S3).

The predictive capacity of the FIB-4 index in the full sample was confirmed for both the 1.3 (HR 1.382 (95% CI [1.064; 1.796], p = 0.015) and 2.0 (HR 1.978 (95% CI [1.632; 2.398], p < 0.0001) cutoff. When comparing participants with a FIB-4 index ≥2.67 to those with a FIB-4 index <1.3, a HR of 2.773 (95% CI [1.937; 3.968], p < 0.0001) was obtained (Fig. 4a, Supplementary Figure S7, and Supplementary Table S4). The HRs in function of the FIB-4 index are provided in Supplementary Figure S8.

The constituents of the FIB-4 index (age, platelets, AST, and ALT), were also predictive of all-cause mortality, with HRs of 1.069 (95% CI [1.058; 1.080], p < 0.0001) for age, 0.998 (95% CI [0.996; 0.999], p = 0.0071) for platelets (1/nl), 1.009 (95% CI [1.003; 1.014], p = 0.0010) for AST (U/l) and 0.992 (95% CI [0.986; 0.999], p = 0.024) for ALT (U/l) (Supplementary Table S5).

As an explorative analysis, we dissected the predictive capacity of the FIB-4 index on all-cause mortality in the different HF phenotypes. The FIB-index was predictive for all-cause mortality in all HF phenotypes (Supplementary Figure S9A). Using a model adjusted for age and sex (Supplementary Figure S9B), the FIB-4 index on a continuous scale predicted all-cause mortality in patients with HF with reduced ejection fraction (HFrEF) with an FLI of at least 60, but not in those with an FLI below 60. This trend was also present in patients with HF with midrange ejection fraction (HFmrEF) in which the FIB-4 index was only predictive for all-cause mortality in individuals with an FLI of at least 60. In contrast, the FIB-4 index was predictive for all-cause mortality across all FLI categories in patients with HFpEF with the highest HR observed in individuals with an FLI below 30.

## Discussion

It has been hypothesized that hepatic inflammation from MASLD can contribute to the cardiac phenotype in the context of metabolic inflammation.^3^^,^^12^ On the other hand, cardiac congestion can produce hepatic fibrosis over time.^3^^,^^8^ Employing the FIB-4 index, a surrogate and inexpensive marker of hepatic fibrosis, could facilitate the estimation of cardiac and hepatic reserve in both primary and specialty care and hence promote the multidisciplinary management of patients with heart failure and liver fibrosis or hepatic venous congestion.^3^^,^^20^

The FIB-4 index has been earlier found to predict all-cause mortality in patients admitted with decompensated chronic HF.^11^ Considering the pleiotropic origins of hepatic fibrosis potentially involved in HF along with the high prevalence of MASLD^13^ it is imperative to dissect the effect of liver fibrosis on HF against the relevant pathophysiological basis.

We found that the FIB-4 index predicted all-cause mortality in patients with chronic HF. Although the constituents of the FIB-4 index were also predictive of all-cause mortality in the study sample, their combination is less subject to alterations in one specific parameter and incorporates different pathophysiological processes.^16^ In that regard, a transient AST elevation originating from myocardial infarction, which is a common cause of heart failure,^21^ can influence the FIB-4 index, but AST levels generally return to normal after approximately 7–10 days.^22^

The relation between the FIB-4 index and all-cause mortality was the strongest in participants without MASLD which was confirmed by an interaction analysis. This finding was reflected by the potent association between the FIB-4 index and NT-proBNP levels in study participants with an FLI below 60, since NT-proBNP levels, apart from HF, as well relate to cardiovascular and all-cause mortality.^23^

In contrast, regression of hepatic steatosis determined by FLI categories, has been found to decrease the risk for incident HF, hospitalization for HF, and liver-related mortality^24^ and it was recently reported that hepatic steatosis assessed by magnetic resonance imaging proton density fat fraction, independently predicts a higher heart rate and supports early cardiac remodeling with reduced ventricular volumes in the general population.^25^ The fact that MASLD predicts incident HF,^4^ but also blunts the association between liver fibrosis and NT-proBNP levels and its ability to predict mortality, suggests that MASLD can promote HF development because of metabolic inflammation, but once cardiac changes have occurred, congestive hepatopathy resulting from HF could take over the prognosis. A potential role of venous congestion^26^ in the predictive ability of the FIB-4 index in HF was further supported by relevant associations with cardiac hemodynamic functions including systolic and diastolic dysfunction, in those having no MASLD. Furthermore, significant associations were observed between the FIB-4 index and LVMI, as a measure of left ventricle hypertrophy, only in patients without MASLD. Another factor that could explain the predictive ability of the FIB-4 index for mortality and its association with cardiac dysfunction in the absence of MASLD is increased portal pressure in advanced congestive hepatopathy, which adds hemodynamic failure to the already existing relative hypovolemia in HF.^26^, ^27^, ^28^ As a result, hepatic fibrosis in the absence of MASLD could result from long-lasting venous congestion, while the first triggers for cardiac remodeling could have had their basis in metabolic inflammation from MASLD.^2^^,^^12^ On the other hand, hepatic venous congestion could exacerbate MASLD-driven liver fibrogenesis, resulting in higher FIB-4 indices.^23^ The predictive capacity of the FIB-4 index in HF-mediated congestive hepatopathy should therefore be placed in the context of MASLD and could therefrom be a convenient tool for estimating combined hepatic and cardiac reserve.^8^

On the other hand, intrahepatic fat content decreases upon increasing fibrosis stage, which can be an alternative explanation for the increasing predictive ability of the FIB-4 index with decreasing FLI.^29^ Moreover, patients with cirrhosis and less than 33% hepatic fat have a shorter survival and increased risk of liver-related complications when compared to patients with cirrhosis and at least 33% hepatic fat, which could relate to liver dysfunction and malnutrition. This concept can originate from a catabolic state in which liver fat is mobilized to fulfill metabolic needs, a process coined ‘burnt-out MASH’.^30^ Although patients with MASH have a high level of systematic inflammation and often poor cardiovascular health,^3^^,^^12^ the stage of liver fibrosis remains the most important predictor of mortality.^31^

Nonetheless, the FIB-4 index was predictive of all-cause mortality in all FLI categories, suggesting that the FIB-4 index can also be used in patients with heart failure without first assessing MASLD.

As age is a confounding factor on the FIB-4 index,^32^ we assessed apart from the 1.3 cutoff, also the 2.0 cutoff in the full sample, which were both predictive of all-cause mortality in the study sample. Nevertheless, since hepatic fibrosis produced from congestive hepatopathy with different MASLD stages constitute specific phenotypes,^8^^,^^26^ it remains to be determined which cutoffs can be best employed for specific subgroups.

The results of this study must be interpreted in the context of its limitations. First, bias could exist regarding liver disease etiology. Although patients consuming significant amounts of alcohol were excluded from the analysis, it was a self-reported parameter, risking recall bias. Further, patients suffering from viral hepatitis were not excluded from the analysis, although the prevalence of both hepatitis B and C are low in Germany,^33^ in particular when compared to the burden of alcohol-related liver disease^34^ and MASLD.^6^ Second, it was impossible to differentiate between liver fibrosis and congestive hepatopathy. Third, the role of age as cofactor on disease progression and the FIB-4 index, and the FIB-4 index and FLI as surrogate scores for liver fibrosis and MASLD, respectively, are sources of bias.^3^^,^^32^ Fourth, the sample sizes of the HF phenotypes in the explorative longitudinal analysis were relatively small for HFrEF and HFmrEF. Last, the study sample consisted mainly of patients of white ancestry in a Western-European setting, limiting the external validity. On the other hand, the study consisted of a large well-defined cohort spanning the full HF spectrum. In addition, the follow-up time of nine years was long enough for making accurate real-world estimations in the longitudinal analysis since the five-year survival rate of patients with HF is approximately 59.7%.^35^ Furthermore, the results from the longitudinal analysis were in line with the results obtained from the cross-sectional multivariable linear regression analyses regarding NT-proBNP levels and measures of cardiac function and structure.

### Conclusions

These data indicate that the FIB-4 index can be a readily accessible NIT for risk assessment in HF, also in less resourced settings including primary care. Future strategies for cardiovascular disease management should take into account the prognostic value of liver fibrosis, of which those without MASLD tend to have larger cardiac functional and structural changes and are hence especially at risk for mortality.

## Contributors

Conceptualization: JB, JP, PSW, JMS. Methodology: all authors. Software: JB, AG, JMS. Validation: JB, AG, JMS. Formal analysis: JB, AG. Investigation: all authors. Resources: JP, PSW, JMS. Data Curation: JB, AG, JMS. Writing–Original Draft: JB, JMS. Writing–Review & Editing: all authors. Visualization: JB, AG. Supervision: PSW, JMS. Project administration: JP, PSW, JMS. Funding acquisition: JP, PSW.

## Data sharing statement

Research data will be shared with academic researchers who submit a research proposal approved by an independent review board. Individual patient data will be shared in a de-identified and anonymized format. Data will be made available upon publication. Information about the study and requesting research data related to this study can obtained by contacting the corresponding author (joern.schattenberg@uks.eu) and visiting/www.unimedizin-mainz.de/pkmp-cesm/research-studies/studies-bio-databases/myovasc.html?L=1.

## Declaration of interests

**J.B.** reports outside the submitted work research funding from Colgate-Palmolive.

**J.P.** reports personal fees from Bayer AG, Daiichi-Sankyo, and Sanofi-Aventis. He is an employee of Boehringer Ingelheim International GmbH, but was not employed at the time of conception and design of the study and during analysis and interpretation of data.

**M.B.** reports research support from the Deutsche Forschungsgemeinschaft, speakers honoraria from Abbott, Amgen, Astra Zeneca, Bayer, Boehringer-Ingelheim, Bristol Myers Squibb, Cytokinetics, Medtronic, Novartis, Servier, and Vifor. He is or was part of the advisory board for Amgen, Bayer, Boehringer Ingelheim, Cytokinetics, Medtronic, Novartis, Pfizer, ReCor, Servier, and Vifor.

**P.S.W** reports outside the submitted work: consulting fees from Astra Zeneca, research grants from Bayer AG, research grants, consulting and lecturing fees from Bayer Health Care, lecturing fees from Bristol Myers Squibb, grants and consulting fees from Boehringer Ingelheim, grants, consulting and lecturing fees from Daiichi Sankyo Europe, consulting fees and non-financial support from Diasorin, non-financial support from I.E.M., grants and consulting fees from Novartis Pharma, lecturing fees from Pfizer Pharma, non-financial grants from Philips Medical Systems, and grants and consulting fees from Sanofi-Aventis.

**J.M.S.** has acted as Consultant to Apollo Endosurgery, Albireo Pharma Inc, Bayer, Boehringer Ingelheim, Gilead Sciences, GSK, Intercept Pharmaceuticals, Ipsen, Inventiva Pharma, Madrigal, MSD, Northsea Therapeutics, Novartis, Novo Nordisk, Pfizer, Roche, Sanofi, Siemens Healthineers; has received research funding from Gilead Sciences, Boehringer Ingelheim, Siemens Healthcare GmbH and Speaker Honorarium from Boehringer Ingelheim, Echosens, MedPublico GmbH, Novo Nordisk, Madrigal Pharmaceuticals.

**A.G.** reports no potential conflict of interest.

## Appendix A Supplementary data

Supplementary material

Renamed_76131

STROBE Checklist
