# Supplementary material for: Clinical utility of the Fibrosis-4 index for predicting mortality in patients with heart failure with or without metabolic dysfunction-associated steatotic liver disease: a prospective cohort study
Source: Lancet Reg Health Eur. 2024 Nov 30;48:101153. doi: 10.1016/j.lanepe.2024.101153 (PMC11648889; doi:10.1016/j.lanepe.2024.101153)
Supplement: Supplementary material [file mmc1.docx]

**Supplementary material**

**Clinical utility of the Fibrosis-4 index for predicting mortality in patients with heart failure in the context of metabolic dysfunction-associated steatotic liver disease: a prospective cohort study**

Joost Boeckmans, Jürgen H Prochaska, Alexander Gieswinkel, Michael Böhm, Philipp S Wild^*^, and Jörn M Schattenberg^*^

*Senior authors

**Table of contents**

Definitions of diseases……….…….……….Page 2

Supplementary figures…….………………..Page 3

Supplementary tables………………………Page 12

**Definitions of diseases**

- Arterial hypertension: systolic:diastolic blood pressure ≥140:90 mmHg or physician-diagnosed arterial hypertension.
- Diabetes mellitus: diagnosed by a physician, use of antidiabetic drugs (ATC-Code: A10A and B) or HbA1c ≥6.5 %.
- Obesity: Body-Mass-Index ≥30 kg/m2.
- Dyslipidemia: diagnosed by a physician, LDL/HDL-ratio >3.5, triglyceride level >150 mg/dl in the fasting state, or intake of lipid-modifying drugs (ATC-code C10).
- Smoking: at least one cigarette per day.
- Family history of myocardial infarction or stroke: myocardial infarction or stroke in a male first-degree relative ≤60 years or in a female first-degree relative ≤65 years.
- Chronic kidney disease: estimated glomerular filtration rate <60ml/min/1.73m² (Chronic Kidney Disease Epidemiology Collaboration formula)
- Cancer: based on the question “Was there ever a diagnosis of cancer in your life?”.
- Other comorbidities were retrieved using medical records and computer-assisted interviews.

**Definitions of medication** (Anatomical Therapeutic Chemical (ATC) codes)

Antidiabetic medication: ATC A10

Lipid-modifying agents: ATC C10

Agents acting on the RAS: ATC C09

Beta blockers: ATC C07

Calcium channel blocker: ATC C08

Digitalis glycosides, anti-arrhythmics, and vasodilators: ATC C01

Antithrombotic agents: ATC B01A

**Supplementary figure 1: Schoenfeld test for Cox proportional hazards analysis (Fibrosis-4 index on continuous scale).**

**Supplementary figure 2: Assumption check for linear regression log(NT-proBNP) [SD] - FIB-4 index.** [abbreviations: FIB-4: fibrosis-4; NT-proBNP, N-terminal pro-B-type natriuretic peptide]


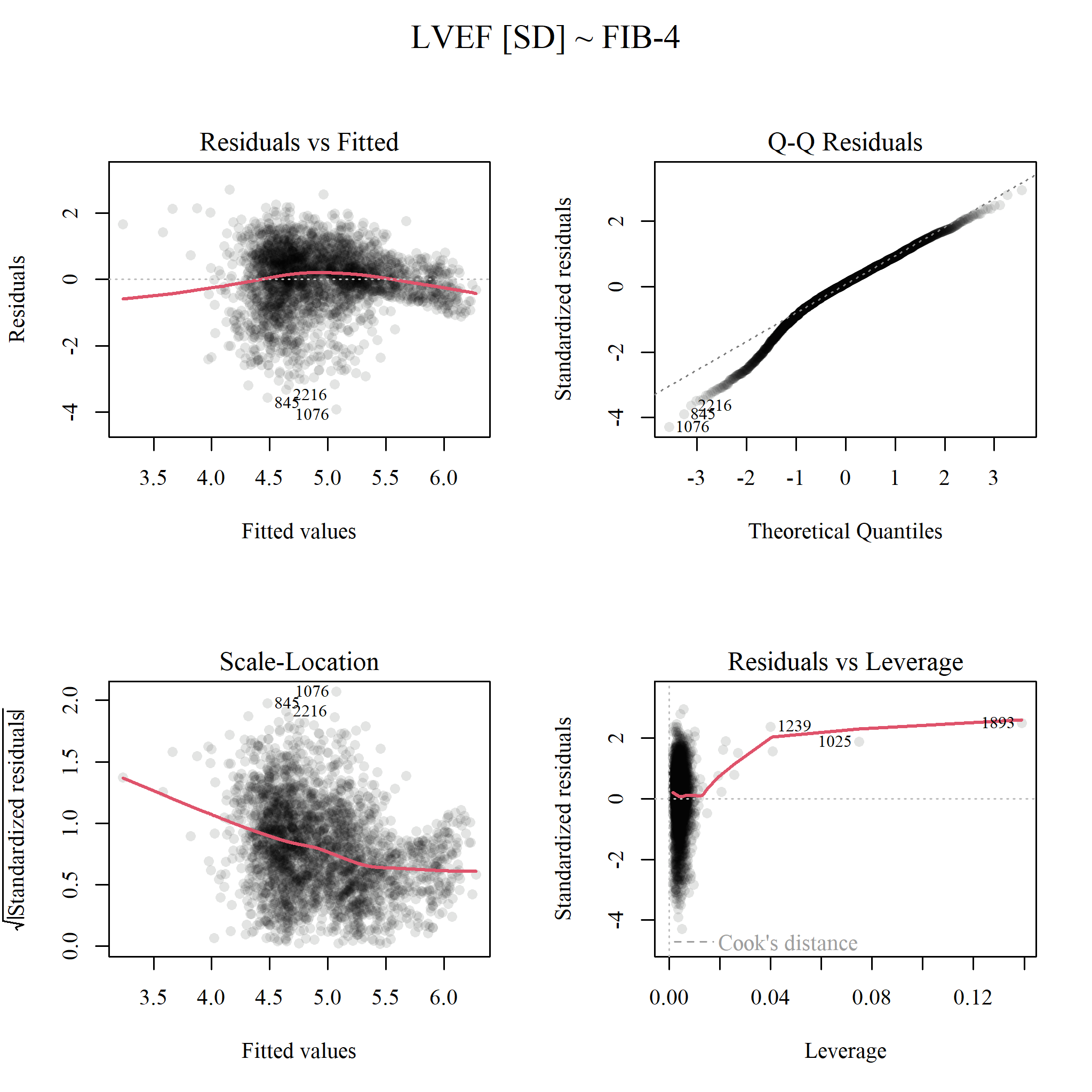


**Supplementary figure 3: Assumption check for linear regression LVEF [SD] - FIB-4 index.** [abbreviations: FIB-4: fibrosis-4; LVEF, left ventricular ejection fraction]


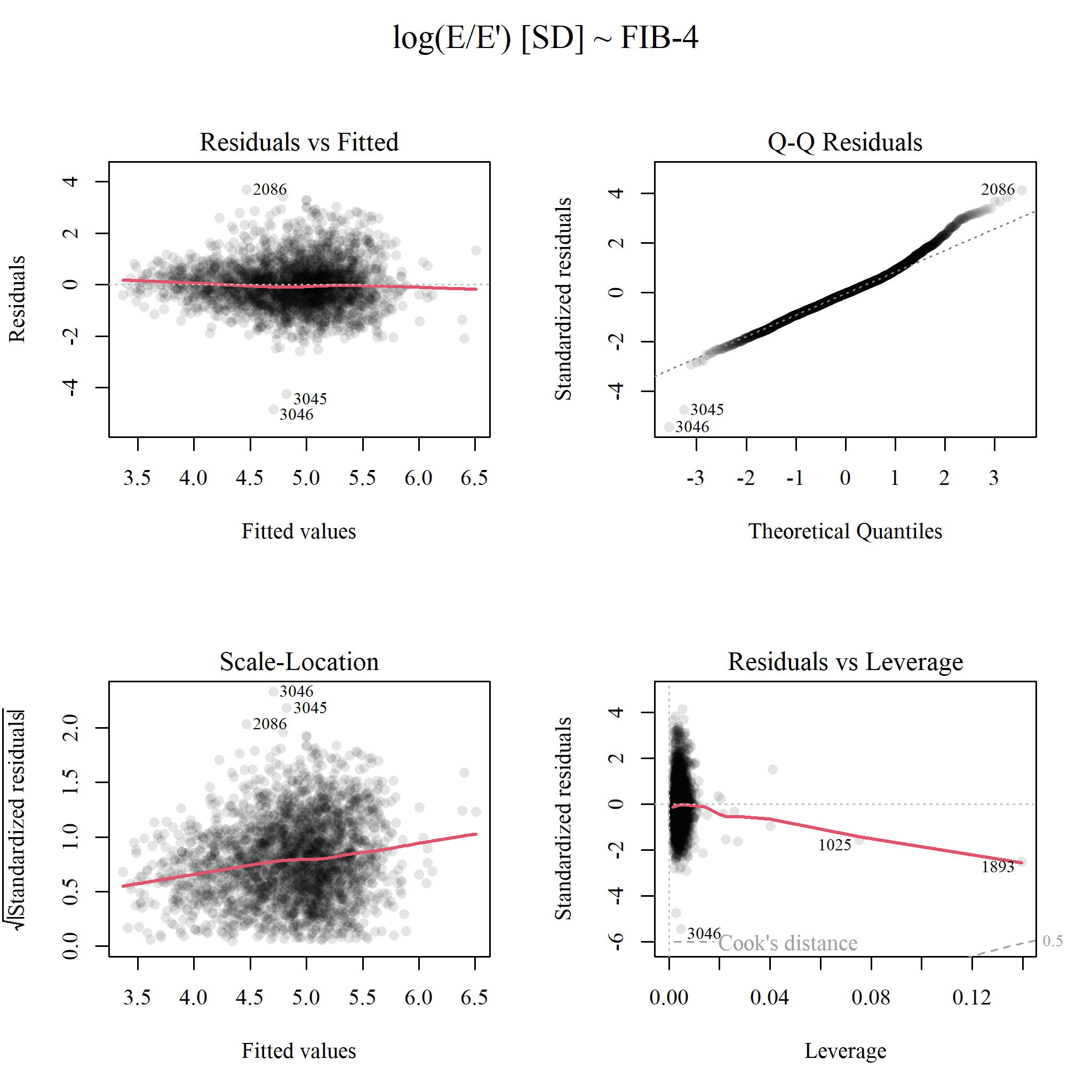


**Supplementary figure 4: Assumption check for linear regression log(lateral E/E’ ratio) [SD] - FIB-4 index.** [abbreviations: FIB-4: fibrosis-4]


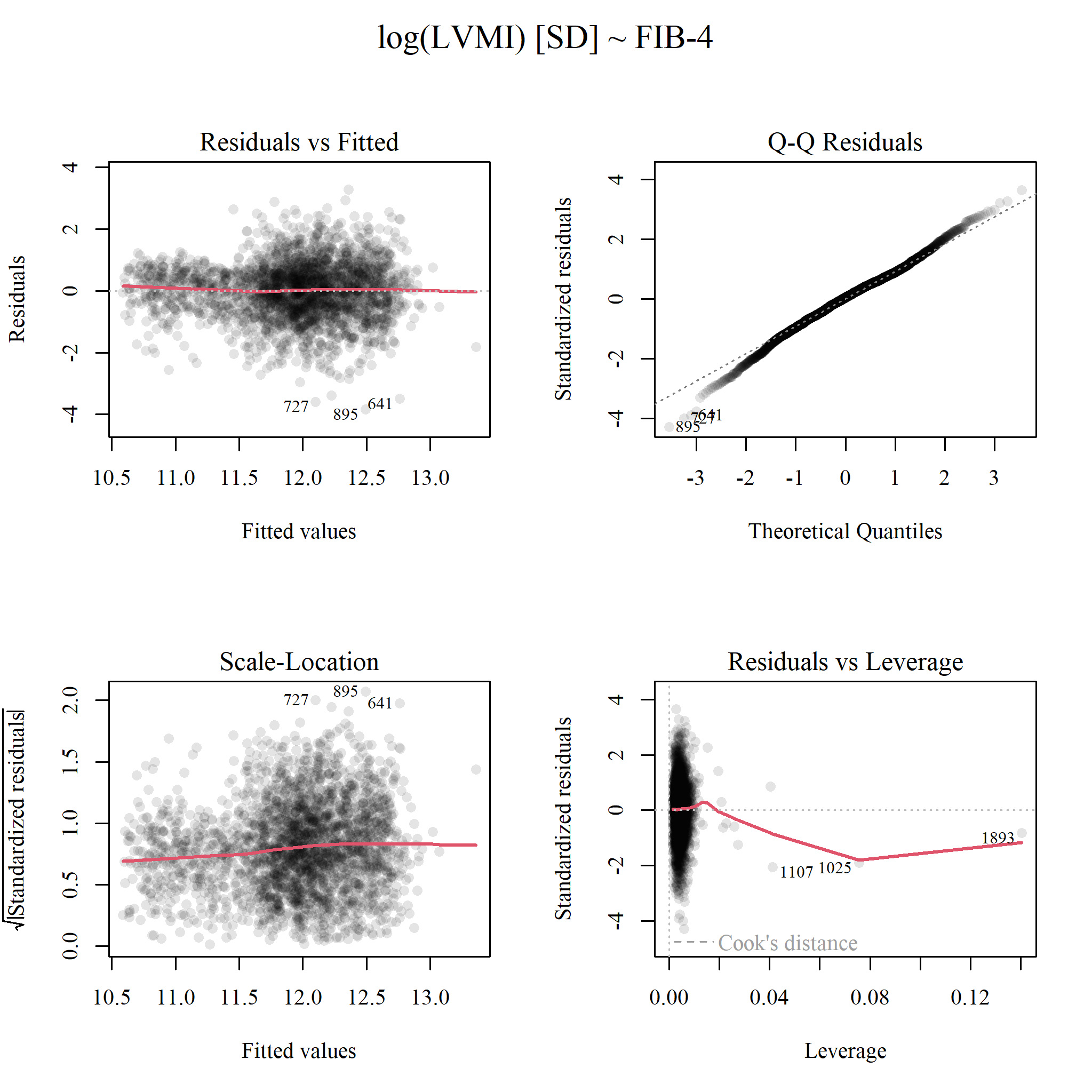


**Supplementary figure 5: Assumption check for linear regression log(LVMI) [SD] - FIB-4 index.** [abbreviations: FIB-4: fibrosis-4; LVMI, left ventricular mass indexed to height^2.7^]


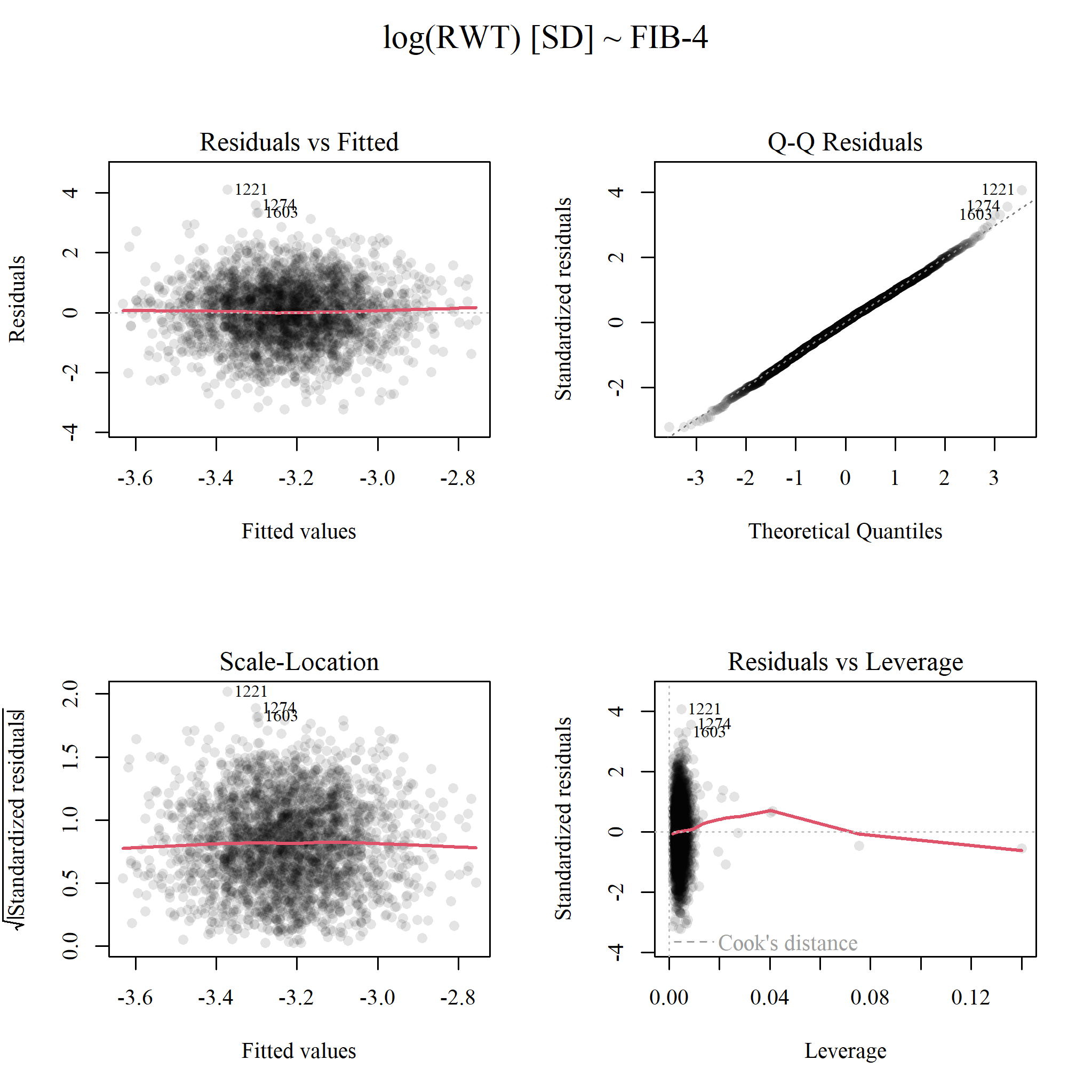


**Supplementary figure 6: Assumption check for linear regression log(RWT) [SD] - FIB-4 index.** [abbreviations: FIB-4: fibrosis-4; RWT, relative wall thickness]


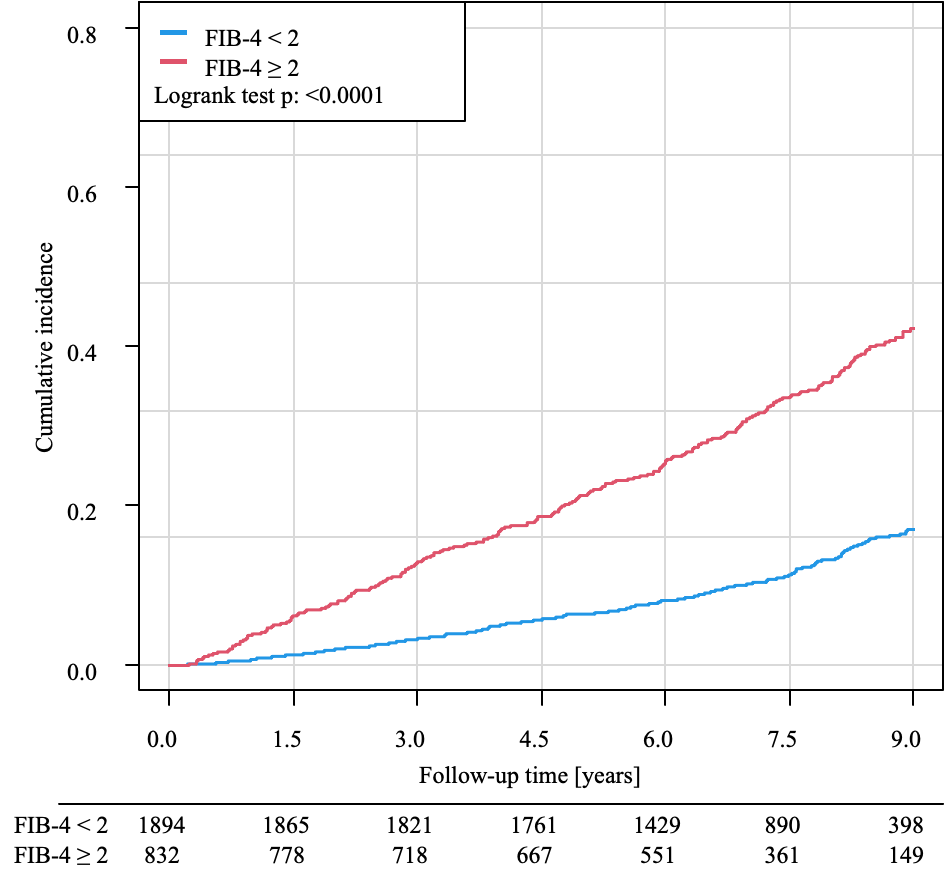


**Supplementary figure 7: Longitudonal relationship between the FIB-4 index and all-cause mortality.** Red line denotes study participants with FIB-4 index ≥ 2, blue line denotes study participants with FIB-4 index < 2. Level of significance: p < 0.05 is considered as statistically significant (Kaplan-Meier analysis with log rank test) (y-axis: cumulative incidende; x-axis: time in years). [abbreviations: FIB-4: fibrosis-4]


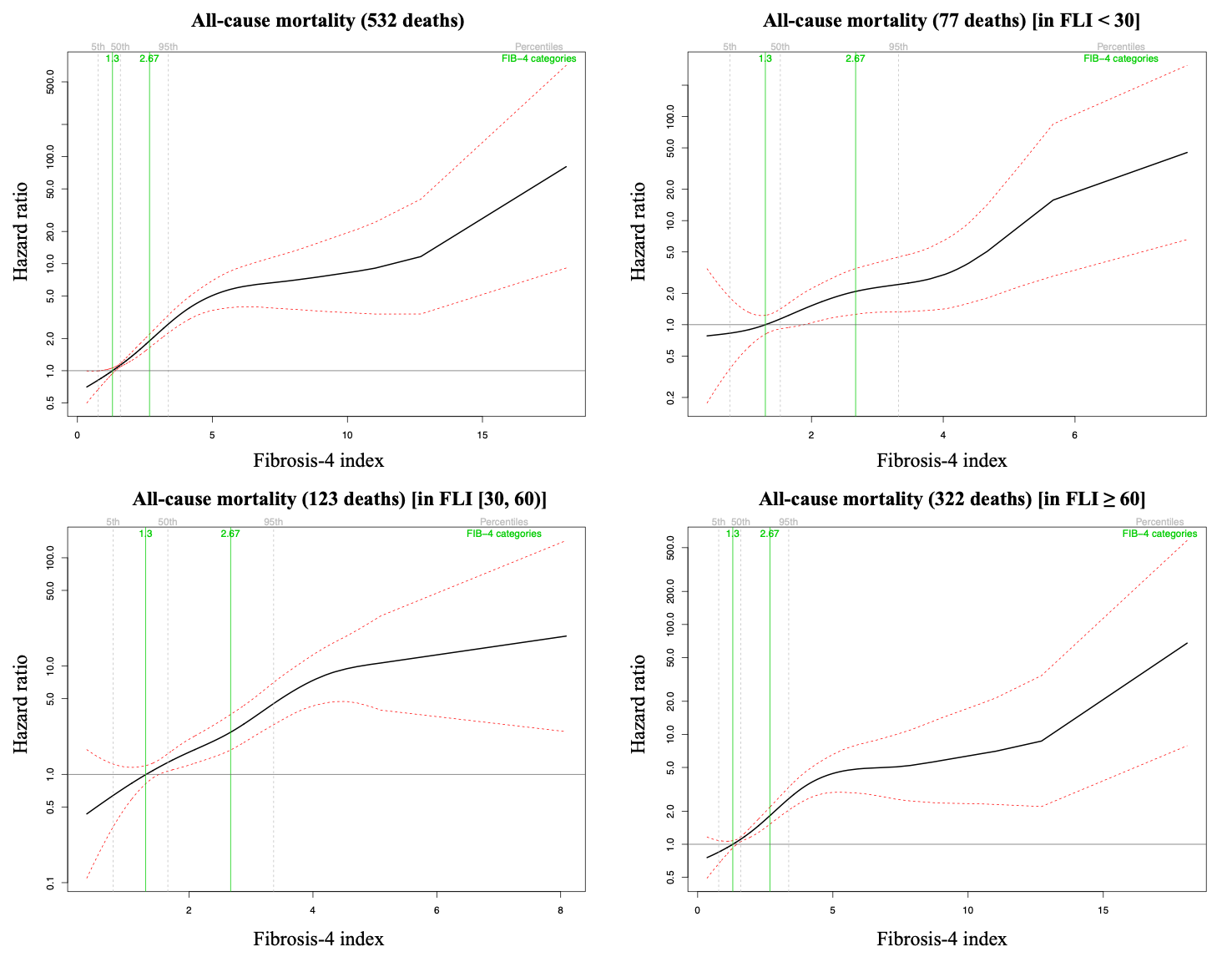


**Supplementary figure 8: Hazard ratios in function of the fibrosis-4 index in the whole sample and stratified by FLI.** (FLI missing for 10 study participants).

**Supplementary figure 9: Longitudonal relationship between the FIB-4 index and all-cause mortality in heart failure phenotypes stratified by FLI categories.** (A) All-cause mortality in patients with HFpEF, HFmrEF and HFrEF according to the FIB-4 index and stratified by FLI categories (color lines indicate cumulative incidences based on FIB-4 index category). Level of significance: p < 0.05 is considered as statistically significant (Kaplan-Meier analysis with log rank test) (y-axis: cumulative incidende; x-axis: time in years). (B) Longitudinal analysis for all-cause mortality in heart failure phenotypes according to the FIB-4 index (continuous scale) and stratified by FLI categories (symbols represent hazard ratios and bars represent 95% confidence intervals). Level of significance: p < 0.05 is considered as statistically significant (Cox regression; age- and sex-adjusted). [abbreviations: CI: confidence interval, FIB-4: fibrosis-4; HFmrEF: heart failure with midrange ejection fraction; HFpEF: heart failure with preserved ejection fraction; HFrEF: heart failure with reduced ejection fraction; FLI: fatty liver index]

| **Supplementary table 1: missing values at baseline.** | |
| --- | --- |
| Sex | 0% (0/2726) |
| Age [y] | 0% (0/2726) |
| Body mass index [kg/m²] | 0.37% (10/2726) |
| Systolic blood pressure [mmHg] | 0.18% (5/2726) |
| Diastolic blood pressure [mmHg] | 0.18% (5/2726) |
| Heart rate [bpm] | 1.43% (39/2726) |
| Fatty liver index | 0.95% (26/2726) |
| Fibrosis-4 index | 0% (0/2726) |
| Arterial hypertension | 0% (0/2726) |
| Diabetes mellitus | 0% (0/2726) |
| Smoking | 0% (0/2726) |
| Obesity | 0% (0/2726) |
| Dyslipidemia | 0% (0/2726) |
| Family history of myocardial infarction /stroke | 0.04% (1/2726) |
| History of myocardial infarction | 0% (0/2726) |
| History of stroke | 0% (0/2726) |
| Coronary artery disease | 0% (0/2726) |
| Atrial fibrillation | 0% (0/2726) |
| Peripheral artery disease | 0% (0/2726) |
| Chronic kidney disease* | 0% (0/2726) |
| Cancer | 0% (0/2726) |
| Cholesterol [mmol/l] | 0.11% (3/2726) |
| High-density lipoprotein [mg/dl] | 0.22% (6/2726) |
| Low-density lipoprotein [mg/dl] | 1.69% (46/2726) |
| Triglycerides [mg/dl] | 0.11% (3/2726) |
| Estimated glomerular filtration rate [ml/min/1.73m2] | 0% (0/2726) |
| Aspartate aminotransferase [U/l] | 0% (0/2726) |
| Alanine aminotransferase [U/l] | 0% (0/2726) |
| HbA1c [%] | 0.11% (3/2726) |
| Glucose [mg/dl] | 0.04% (1/2726) |
| N-terminal pro–B-type natriuretic peptide [pg/ml] | 0.88% (24/2726) |
| C-reactive protein [mg/L] | 1.50% (41/2726) |
| Left ventricular ejection fraction [%] | 0% (0/2726) |
| Left ventricular lateral E/E' ratio | 0.55% (15/2726) |
| Left ventricular mass [g] | 1.39% (38/2726) |
| Left ventricular mass/height [g/m^2·7^] | 1.65% (45/2726) |
| Relative wall thickness | 1.36% (37/2726) |
| Symptomatic HF (Stage C-D) | 6.31% (172/2726) |
| Heart failure with preserved ejection fraction | 0% (0/2726) |
| Heart failure with midrange ejection fraction | 0% (0/2726) |
| Heart failure with reduced ejection fraction | 0% (0/2726) |
| Antidiabetic medication (A10) | 0% (0/2726) |
| Lipid-modifying agents (C10) | 0% (0/2726) |
| Agents acting on the RAS (C09) | 0% (0/2726) |
| Beta blockers (C07) | 0% (0/2726) |
| Calcium channel blocker (C08) | 0% (0/2726) |
| Digitalis glycosides, anti-arrhythmics, and vasodilators (C01) | 0% (0/2726) |
| Antithrombotic agents (B01A) | 0% (0/2726) |

| **Supplementary table 2:** **Distribution of heart failure based on the FIB-4 index and stratified by FLI.**  Data presented as relative and absolute frequencies. | | | | |
| --- | --- | --- | --- | --- |
| **Variable** | **All (2726)** | **Stage 0/A (471)** | **Stage B/C/D (2255)** | **p-value** |
|  |  |  |  |  |
| **FIB-4 index** | | | | |
| FIB-4 < 1.3 | 32.8% (n: 894/2726) | 55.2% (n: 260/471) | 28.1% (n: 634/2255) | **<0.0001** |
| FIB-4 1.3 to 2.67 | 53.9% (n: 1468/2726) | 42.3% (n: 199/471) | 56.3% (n: 1269/2255) | **<0.0001** |
| FIB-4 ≥ 2.67 | 13.4% (n: 364/2726) | 2.5% (n: 12/471) | 15.6% (n: 352/2255) | **<0.0001** |
|  |  |  |  |  |
| **FIB-4 index with FLI < 30** | | | | |
| FIB-4 < 1.3 | 8.9% (n: 242/2718) | 23.9% (n: 112/469) | 5.8% (n: 130/2249) | **<0.0001** |
| FIB-4 1.3 to 2.67 | 12.8% (n: 346/2711) | 16.8% (n: 79/470) | 11.9% (n: 267/2241) | **0.0049** |
| FIB-4 ≥ 2.67 | 2.8% (n: 75/2723) | 0.2% (n: 1/471) | 3.3% (n: 74/2252) | **<0.0001** |
|  |  |  |  |  |
| **FIB-4 index with FLI 30 to 60** | | | | |
| FIB-4 < 1.3 | 7.6% (n: 206/2718) | 14.7% (n: 69/469) | 6.1% (n: 137/2249) | **<0.0001** |
| FIB-4 1.3 to 2.67 | 15.0% (n: 406/2711) | 10.6% (n: 50/470) | 15.9% (n: 356/2241) | **0.0035** |
| FIB-4 ≥ 2.67 | 3.5% (n: 94/2723) | 0.6% (n: 3/471) | 4.0% (n: 91/2252) | **<0.0001** |
|  |  |  |  |  |
| **FIB-4 index with FLI ≥ 60** | | | | |
| FIB-4 < 1.3 | 16.1% (n: 438/2718) | 16.4% (n: 77/469) | 16.1% (n: 361/2249) | 0.84 |
| FIB-4 1.3 to 2.67 | 25.9% (n: 701/2711) | 14.7% (n: 69/470) | 28.2% (n: 632/2241) | **<0.0001** |
| FIB-4 ≥ 2.67 | 7.1% (n: 192/2723) | 1.7% (n: 8/471) | 8.2% (n: 184/2252) | **<0.0001** |

Abbreviations: FIB-4: fibrosis-4; FLI: fatty liver index.

| **Supplementary table 3: Interaction terms for the primary outcome of all-cause mortality: FIB-4 index according to FLI categories**  [abbreviations: CI: confidence interval; FIB-4: fibrosis-4; HR: hazard ratio]  Level of significance: a p-value < 0.05 is considered as statistically significant.  Adjustments:  Model 1: adjustment for FLI categories (binary), age, and sex (binary),  Model 2: additional adjustment for arterial hypertension (binary), diabetes mellitus (binary), smoking (binary), obesity (binary), dyslipidemia (binary), and family history of myocardial infarction/stroke (binary);  Model 3: additional adjustment for cancer (binary), atrial fibrillation (binary), chronic kidney disease (binary), coronary artery disease (binary), myocardial infarction (binary), peripheral artery disease (binary), and stroke (binary). | | | | |
| --- | --- | --- | --- | --- |
| **Model 1 (C-index 0.7241; 522 events, N = 2,700)** | | | | |
| All-cause mortality ~ | **HR - interaction term** | **L 95% CI** | **U 95% CI** | **p-value** |
| FIB-4 index and FLI < 30 (reference) | 1.711 | 1.358 | 2.157 | **<0.0001** |
| FIB-4 index and FLI 30 - 60 | 1.016 | 0.775 | 1.333 | 0.91 |
| FIB-4 index and FLI ≥ 60 | 0.766 | 0.604 | 0.970 | **0.027** |
| **Model 2 (C-index 0.7379; 522 events, N = 2,699)** | | | | |
| All-cause mortality ~ | **HR - interaction term** | **L 95% CI** | **U 95% CI** | **p-value** |
| FIB-4 index and FLI < 30 (reference) | 1.664 | 1.333 | 2.077 | **<0.0001** |
| FIB-4 index and FLI 30 - 60 | 1.015 | 0.783 | 1.317 | 0.91 |
| FIB-4 index and FLI ≥ 60 | 0.774 | 0.617 | 0.972 | **0.027** |
| **Model 3 (C-index 0.7583; 522 events, N = 2,699)** | | | | |
| All-cause mortality ~ | **HR - interaction term** | **L 95% CI** | **U 95% CI** | **p-value** |
| FIB-4 index and FLI < 30 (reference) | 1.658 | 1.330 | 2.066 | **<0.0001** |
| FIB-4 index and FLI 30 - 60 | 0.949 | 0.732 | 1.230 | 0.69 |
| FIB-4 index and FLI ≥ 60 | 0.766 | 0.611 | 0.961 | **0.021** |

| **Supplementary table 4: Cox-regression analysis (all-cause mortality) for Fibrosis-4 index categories**  [abbreviations: AST, aspartate aminotransferase; ALT, alanine aminotransferase CI: confidence interval; FIB-4: fibrosis-4; HR: hazard ratio]  Level of significance: a p-value < 0.05 is considered as statistically significant.  Adjustments:  Model 1: adjustment for age and sex (binary),  Model 2: additional adjustment for arterial hypertension (binary), diabetes mellitus (binary), smoking (binary), obesity (binary), dyslipidemia (binary), and family history of myocardial infarction/stroke (binary);  Model 3: additional adjustment for cancer (binary), atrial fibrillation (binary), chronic kidney disease (binary), coronary artery disease (binary), myocardial infarction (binary), peripheral artery disease (binary), and stroke (binary). | | | | | | |  |
| --- | --- | --- | --- | --- | --- | --- | --- |
| **Model 1** | | | | | | |  |
|  | **N** | **C-index** | **HR** | **L 95% CI** | **U 95% CI** | **p-value** | |
| All-cause mortality ~ |  |  |  |  |  |  | |
| FIB-4 index ≥ 1.3 / < 1.3 | 2726  (532 events) | 0.6927 | 1.327 | 1.020 | 1.726 | **0.035** | |
| FIB-4 index ≥ 2.67 / < 1.3 | 1258  (246 events) | 0.7746 | 2.552 | 1.779 | 3.662 | **<0.0001** | |
| FIB-4 index ≥ 2.0 / < 2.0 | 2726  (532 events) | 0.7084 | 1.891 | 1.560 | 2.292 | **<0.0001** | |
| **Model 2** | | | | | | |  |
|  | **N** | **C-index** | **HR** | **L 95% CI** | **U 95% CI** | **p-value** | |
| All-cause mortality ~ |  |  |  |  |  |  | |
| FIB-4 index ≥ 1.3 / < 1.3 | 2725  (532 events) | 0.7144 | 1.382 | 1.064 | 1.796 | **0.015** | |
| FIB-4 index ≥ 2.67 / < 1.3 | 1257  (246 events) | 0.7938 | 2.773 | 1.937 | 3.968 | **<0.0001** | |
| FIB-4 index ≥ 2.0 / < 2.0 | 2725  (532 events) | 0.7280 | 1.978 | 1.632 | 2.398 | **<0.0001** | |
| **Model 3** | | | | | | |  |
|  | **N** | **C-index** | **HR** | **L 95% CI** | **U 95% CI** | **p-value** | |
| All-cause mortality ~ |  |  |  |  |  |  | |
| FIB-4 index ≥ 1.3 / < 1.3 | 2725  (532 events) | 0.7441 | 1.321 | 1.017 | 1.716 | **0.037** | |
| FIB-4 index ≥ 2.67 / < 1.3 | 1257  (246 events) | 0.8169 | 2.521 | 1.767 | 3.596 | **<0.0001** | |
| FIB-4 index ≥ 2.0 / < 2.0 | 2725  (532 events) | 0.7528 | 1.852 | 1.526 | 2.246 | **<0.0001** | |

| **Supplementary table 5: Cox-regression analysis (all-cause mortality) for Fibrosis-4 index constituents**  [abbreviations: AST, aspartate aminotransferase; ALT, alanine aminotransferase CI: confidence interval; FIB-4: fibrosis-4; HR: hazard ratio]  Level of significance: a p-value < 0.05 is considered as statistically significant.  Adjustments:  Model 1: adjustment for age and sex (binary),  Model 2: additional adjustment for arterial hypertension (binary), diabetes mellitus (binary), smoking (binary), obesity (binary), dyslipidemia (binary), and family history of myocardial infarction/stroke (binary);  Model 3: additional adjustment for cancer (binary), atrial fibrillation (binary), chronic kidney disease (binary), coronary artery disease (binary), myocardial infarction (binary), peripheral artery disease (binary), and stroke (binary).  For the analyses with age as independent variable, age was not included as a covariate. | | | | | | |  |
| --- | --- | --- | --- | --- | --- | --- | --- |
| **Model 1** | | | | | | |  |
|  | **N** | **C-index** | **HR** | **L 95% CI** | **U 95% CI** | **p-value** | |
| All-cause mortality ~ |  |  |  |  |  |  | |
| Age [y] | 2726  (532 events) | 0.6905 | 1.068 | 1.058 | 1.078 | **0.0001** | |
| Platelets [1/nl] | 2726  (532 events) | 0.6931 | 0.998 | 0.996 | 0.999 | **0.0081** | |
| AST [U/l] | 2726  (532 events) | 0.6952 | 1.010 | 1.005 | 1.015 | **0.00027** | |
| ALT [U/l] | 2726  (532 events) | 0.6925 | 0.993 | 0.987 | 1.000 | **0.047** | |
| **Model 2** | | | | | | |  |
|  | **N** | **C-index** | **HR** | **L 95% CI** | **U 95% CI** | **p-value** | |
| All-cause mortality ~ |  |  |  |  |  |  | |
| Age [y] | 2725  (532 events) | 0.7115 | 1.069 | 1.058 | 1.080 | **<0.0001** | |
| Platelets [1/nl] | 2725  (532 events) | 0.7139 | 0.998 | 0.996 | 0.999 | **0.0071** | |
| AST [U/l] | 2725  (532 events) | 0.7163 | 1.009 | 1.003 | 1.014 | **0.0010** | |
| ALT [U/l] | 2725  (532 events) | 0.7137 | 0.992 | 0.986 | 0.999 | **0.024** | |
| **Model 3** | | | | | | |  |
|  | **N** | **C-index** | **HR** | **L 95% CI** | **U 95% CI** | **p-value** | |
| All-cause mortality ~ |  |  |  |  |  |  | |
| Age [y] | 2725  (532 events) | 0.7428 | 1.056 | 1.045 | 1.067 | **<0.0001** | |
| Platelets [1/nl] | 2725  (532 events) | 0.7431 | 0.998 | 0.997 | 1.000 | **0.032** | |
| AST [U/l] | 2725  (532 events) | 0.7456 | 1.008 | 1.003 | 1.014 | **0.0024** | |
| ALT [U/l] | 2725  (532 events) | 0.7440 | 0.993 | 0.987 | 1.000 | **0.040** | |
