# Supplementary material for: Clinical utility of the Fibrosis-4 index for predicting mortality in patients with heart failure with or without metabolic dysfunction-associated steatotic liver disease: a prospective cohort study
Source: Lancet Reg Health Eur. 2024 Nov 30;48:101153. doi: 10.1016/j.lanepe.2024.101153 (PMC11648889; doi:10.1016/j.lanepe.2024.101153)
Supplement: Renamed_76131 [file mmc2.pdf]

ESC European Society of Cardiology

European Journal of Preventive Cardiology (2021) **28**, 1009–1018
doi:10.1177/2047487320926438

FULL RESEARCH PAPER

Rationale, design and baseline characteristics of the MyoVasc study: A prospective cohort study investigating development and progression of heart failure

Sebastian Göbel[1,2†], Jürgen H. Prochaska[2,3,4†], Sven-Oliver Tröbs[2,3], Marina Panova-Noeva[2,4], Christine Espinola–Klein[1], Matthias Michal[5], Karl J. Lackner[2,6], Tommaso Gori[1,2], Thomas Münzel[1,2,3], and Philipp S. Wild[2,3,4]*

[1]Centre for Cardiology – Cardiology I, University Medical Centre of the Johannes Gutenberg University Mainz, Germany; [2]German Centre for Cardiovascular Research (DZHK), partner site Rhine-Main, Germany; [3]Preventive Cardiology and Preventive Medicine, Centre for Cardiology, University Medical Centre of the Johannes Gutenberg University Mainz, Germany; [4]Centre for Thrombosis and Haemostasis, University Medical Centre of the Johannes Gutenberg University Mainz, Germany; [5]Department of Psychosomatic Medicine and Psychotherapy, University Medical Centre of the Johannes Gutenberg University Mainz, Germany; and [6]Institute for Clinical Chemistry and Laboratory Medicine, University Medical Centre of the Johannes Gutenberg University Mainz, Germany

Received 17 April 2020; accepted 7 January 2020; online publish-ahead-of-print 14 May 2020

Background Heart failure (HF) is a poly-aetiological syndrome with large heterogeneity regarding clinical presentation, pathophysiology, clinical outcome and response to therapy. The MyoVasc study (NCT04064450) is an epidemiological cohort study investigating the development and progression of HF.

Methods The primary objective of the study is (a) to improve the understanding of the pathomechanisms of HF across the full spectrum of clinical presentation, (b) to investigate the current clinical classifications of HF, and (c) to identify and characterize homogeneous subgroups regarding disease development using a systems-oriented approach. Worsening of HF, that is, the composite of transition from asymptomatic to symptomatic HF, hospitalization due to HF, or cardiac death, was defined as the primary endpoint of the study. During a six-year follow-up period, all study participants receive a highly standardized, biannual five-hour examination in a dedicated study centre, including detailed cardiovascular phenotyping and biobanking of various biomaterials. Annual follow-up examinations are conducted by computer-assisted telephone interviews recording comprehensively the participants´ health status, including subsequent validation and adjudication of adverse events.

Results In total, 3289 study participants (age range: 35 to 84 years; female sex: 36.8%) including the full range of HF stages were enrolled from 2013 to 2018. Approximately half of the subjects (n=1741) presented at baseline with symptomatic HF (i.e. HF stage C/D). Among these, HF with preserved ejection fraction was the most frequent phenotype.

Conclusions By providing a large-scale, multi-dimensional biodatabase with sequential, comprehensive medical-technical (sub)-clinical phenotyping and multi-omics characterization (i.e. genome, transcriptome, proteome, lipidome, metabolome and exposome), the MyoVasc study will help to advance our knowledge about the heterogeneous HF syndrome by a systems-oriented biomedicine approach.

* Corresponding author. Philipp S Wild, University Medical Centre of the Johannes Gutenberg University Mainz Langenbeckstr.1, 55131 Mainz, Germany.
Email: philipp.wild@unimedizin-mainz.de

† These authors contributed equally.

Published on behalf of the European Society of Cardiology. All rights reserved. © The Author(s) 2020. For permissions, please email: journals.permissions@oup.com.

Downloaded from https://academic.oup.com/eurjpc/article/28/9/1009/6353474 by guest on 05 September 2024

Trial registration ClinicalTrials.gov; NCT04064450.

Keywords Heart failure • diagnosis • management • prognosis • systems medicine

Introduction

Heart failure (HF) affects 15 million people in Europe and represents the leading cause of hospitalization in individuals aged 65 years and older. The prevalence of HF is increasing, which has been attributed to ageing of the population with a subsequently higher prevalence of predisposing risk factors (e.g. arterial hypertension, type 2 diabetes and obesity), better survival, and more effective treatment of precursors (e.g. myocardial infarction).[1] From a public health perspective, more than 70% of HF-related health care expenditures are explained by hospital admissions.[2] After the diagnosis of HF has been confirmed, survival rates are approximately 50% after five years and 10% after 10 years, resulting in worse survival than in most common types of cancer.[3–6] This is accompanied by a substantial reduction of quality of life in HF patients.[7]

Our current understanding of HF considers it as a clinical syndrome with heterogeneous causes. Since the diagnosis of HF relies on the presence of unspecific symptoms (e.g. shortness of breath) that may be accompanied by clinical signs (e.g. peripheral oedema), diagnosis is challenging and prone to imprecision and misclassification.[8] Current terminology stratifies HF patients into subgroups based upon the assessment of left ventricular ejection fraction (LVEF). Dependent on the classification scheme applied, patients are stratified into HF with reduced EF (HFrEF), HF with preserved EF (HFpEF), and HF with mid-range EF (HFmrEF) or HFpEF with borderline EF, respectively.1,8 Interestingly, there is still no consistency regarding definitions either for HF phenotypes with reduced EF, with cut-off points for EF ranging from 40%[9] to 55%,[10] or for HFpEF.11,12 Based upon current HF classifications, a synthesis of epidemiological surveys suggests that almost half of patients with symptomatic HF in the community suffer from HF with preserved ejection fraction.[13] Although survival is equally poor for both HF phenotypes,[14] the causes of death in HFpEF individuals differ from those with HFrEF.[15] Despite progress in the treatment of HF, its management also remains challenging due to limited knowledge about the pathophysiology of the heterogeneous HF syndrome, limited effectiveness of current preventive strategies, and the progressive nature of HF. Currently, the benefit of medical therapies is limited to patients with HFrEF only, whereas no specific medical therapy is currently approved for patients with HFpEF.[16]

Against this background, the MyoVasc study has been established as a cohort study with highly standardized deep phenotyping for a large, multi-dimensional biodatabase offering the potential to advance our knowledge about the causes, pathophysiology, diagnostics, clinical course and treatment of HF.

Methods and results

Objectives

The MyoVasc study is investigating the development and progression of the HF syndrome, phenotypes of the heterogeneous syndrome, and the interactions of phenotypes with the vasculature regarding their impact on the course of HF. The study programme aims at advancing our knowledge about the heterogeneous HF syndrome by applying a systems-oriented biomedicine approach. The major objective of the project is to improve the medical and biological understanding of HF by investigating worsening of HF in the full continuum state of the disease, ranging from asymptomatic cardiac dysfunction to symptomatic HF.

Specific objectives of the project are (a) to explore clinical and molecular determinants and biological pathways relevant for the development and progression of asymptomatic HF and symptomatic HF, (b) to evaluate the impact of socio-economic, environmental and lifestyle factors ('human exposome') on the clinical course of HF phenotypes, (c) to improve diagnostics of HF, (d) to advance risk stratification for and prediction of specific sequelae and clinical outcome, (e) to define subtypes of HF related to cause and/or outcome, (f) to identify potential novel therapeutic targets for HF phenotypes, and (g) to establish a high-quality, multi-dimensional database as a sustainable resource for HF research.

Study design

The MyoVasc study is an investigator-initiated, prospective, single-centre cohort study with sequential deep clinical phenotyping and biobanking. The study base for the MyoVasc cohort study is mid-western Germany. The flow chart of the MyoVasc study is depicted in *Figure 1*. After recruitment, study participants will be invited for a five-hour baseline examination in the dedicated study centre. During a follow-up period of six years, identical follow-up examinations are performed in the study centre after two, four and six years. Additionally, annual follow-up investigations are performed via detailed computer-assisted telephone interviews (CATI).

Regulatory aspects, ethics and data safety

Approval of the investigational plan and all relevant study documents was obtained from the responsible ethics committees (reference number 837.319.12 (8420-F)) and the data safety commissioner in 2012 before study initiation. Due to the duration of the study and the extent and granularity of the data collection, approval of the study documents and protocol is regularly re-evaluated by these institutions. An ethics and data protection board was initiated to annually re-evaluate the handling of genetic data and reporting of results to study participants within the study and to report the results to the responsible ethics committee for approval. All individuals provided informed written consent prior to study enrolment. All study procedures are designed to ensure that all study staff involved in the study abide by Good Clinical Practice (GCP), Good Epidemiological Practice (GEP) and the ethical principles described in the current revision of the Declaration of Helsinki and the General Data Protection Regulation of the European Union. The study centre is located at the

Downloaded from https://academic.oup.com/eurjpc/article/28/9/1009/6353474 by guest on 05 September 2024

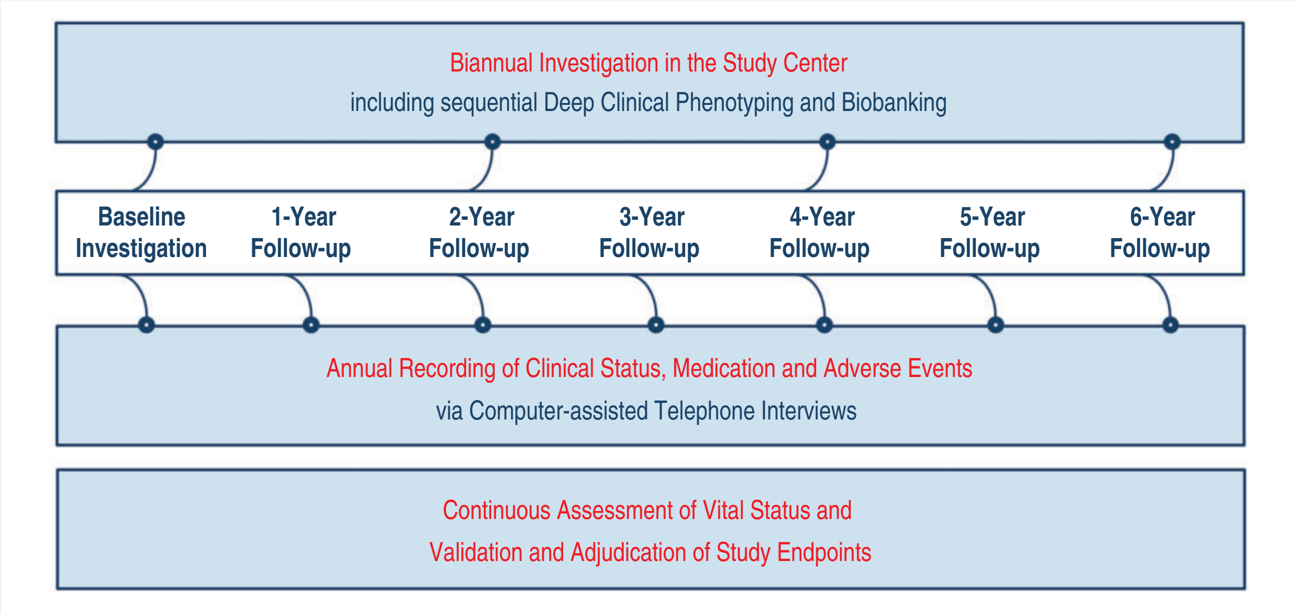

Figure 1 Study flow chart. Vital status is obtained via regular queries to the registration offices. Source data comprise physicians´ letters, medical records and personal communication by study participants or relatives, but also death certificates.

University Medical Centre of the Johannes Gutenberg University in Mainz, Germany. The study programme is led by a steering committee of academic investigators. The MyoVasc study is registered at http://clinicaltrials.gov (identifier: NCT04064450).

Implementation

The MyoVasc study is led by a steering committee of academic investigators. All study investigations are performed by specifically trained staff according to standard operating procedures in a dedicated study centre at the University Medical Centre of the Johannes Gutenberg University Mainz, Germany. A list of the members of the responsible steering committee and the internal study board of the MyoVasc study is provided in the Acknowledgement. An interdisciplinary team with relevant expertise in cardiovascular epidemiology, medicine, biostatistics, bioinformatics and systems biology will make use of the biorepository at many medical, computational and biological levels of analysis.

Inclusion and exclusion criteria

The MyoVasc study includes subjects suffering from systolic or diastolic cardiac dysfunction or HF. Further inclusion criteria were: (a) age 35 to 84 years and (b) sufficient knowledge of the German language to understand study documents and computer-assisted interviews. In order to reflect the full spectrum of HF, patients at all stages of HF (i.e. individuals at American College of Cardiology/American Heart Association (ACC/AHA) HF Stages A–D) were eligible for study enrolment to the HF cohort. Exclusion criteria were: (a) acute endocarditis, myocarditis or pericarditis within the last six months prior to inclusion; (b) acute myocardial infarction within the last three months prior to inclusion (in the case of non-ST-segment-elevation-myocardial infarction) or within the last four months prior to inclusion (in the case of ST-segment-elevation myocardial infarction); (c) acute infectious disease; (d) acute decompensated HF; (e) inability to give written consent.

Recruitment

For the recruitment of the HF study sample, in- and outpatients of the University Medical Centre Mainz with cardiac dysfunction or prevalent HF were contacted by the staff of the MyoVasc study centre. Potential study participants were informed in detail about the study, and eligibility according to the inclusion and exclusion criteria was checked. If the patient was willing to participate, the patient received the study information brochure and an appointment for study enrolment, and the subsequent baseline examination in the MyoVasc study centre was done. Analysis of these items will allow an estimate of the size and direction of potential selection bias.

For the recruitment of the control group, different recruitment modalities have been chosen. A sample of 10,000 men and women was drawn at random from official inhabitant data files. Individuals drawn in the sample from the official inhabitant data files were invited to participate in the study. If the study team received positive feedback, potential study participants were contacted to review the inclusion and exclusion criteria. If echocardiography performed in the study centre revealed normal function, individuals were included in the control group. If cardiac dysfunction was identified, individuals were included in the HF study sample as individuals at risk for developing symptomatic HF.

Outcome

The primary outcome is defined specifically for each sample of the MyoVasc study (*Figure 2*). For all study participants with HF, that is, patients with cardiac dysfunction at risk for developing symptomatic HF and patients with symptomatic HF, the primary outcome is worsening of HF. For patients suffering from cardiac dysfunction, the

Downloaded from https://academic.oup.com/eurjpc/article/28/9/1009/6353474 by guest on 05 September 2024

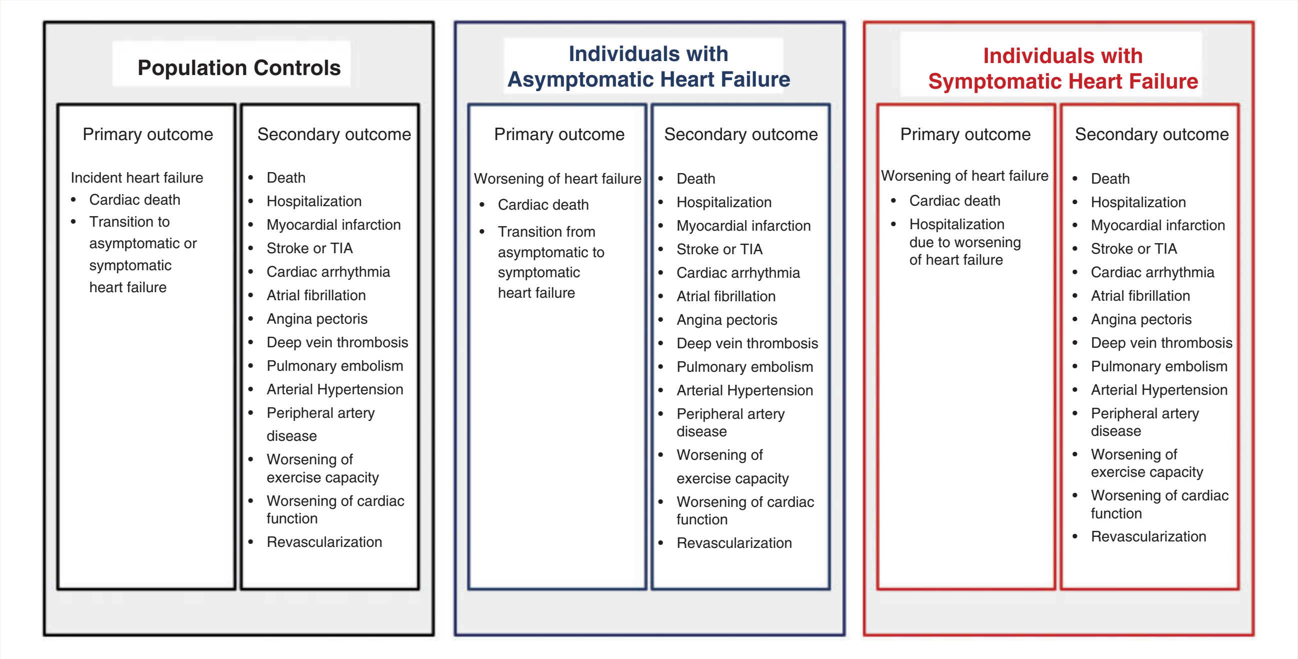

Figure 2 Study outcomes. For all study endpoints, source data (e.g. medical records and death certificates) are obtained for validation and adjudication of outcome data. TIA: transient ischaemic attack.

primary outcome is the composite of (a) cardiac death and (b) the transition to symptomatic HF (i.e. new diagnosis of HF in an ambulatory or inpatient setting). For study participants with symptomatic HF (e.g. subjects with ACC/AHA HF Stages C/D), the primary outcome is the composite of (a) cardiac death and (b) hospitalization due to HF. For the sample of population controls, the primary outcome is the composite of (a) cardiac death and (b) transition to asymptomatic or symptomatic HF. Information on death is obtained via annual CATI and regular checks with the German registration offices. The secondary outcomes are listed in *Figure 2*. All outcome events are defined in a charter for assessment by a clinical event committee. Source data (e.g. death certificates and medical records) will be obtained for all study endpoints for subsequent validation and adjudication by the clinical event committee.

Investigational plan

The set of investigations performed in the study centre are presented in *Table 1* (see Supplemental Appendix for detailed information). All procedures were performed by specifically trained study staff according to standard operating procedures. All subjects were investigated in the same order. Measurement of blood pressure, vascular function and lung function, cardiopulmonary exercise testing and echocardiography were performed in air-conditioned examination rooms at a study centre dedicated to this study. Follow-up investigations by CATI comprise assessment of medication, clinical status, risk factors and comorbidities, and recording of study endpoints (e.g. hospitalization) for subsequent endpoint validation and adjudication (*Table 2*).

Biobanking

A central aim of the MyoVasc study is to establish a biodata bank with multiple qualities of biomaterial including blood samples from study participants at sequential time points. Prior to the investigation at the study centre, subjects are asked to have an overnight fasting period of at least eight hours. A total of 115 ml of venous blood of various qualities (e.g. citrated plasma, EDTA plasma, serum, DNA, RNA, platelet-rich and platelet-poor plasma) and midstream urine was collected at every visit in the study centre according to standard operating procedures with predefined pre-analytical conditions. A comprehensive set of humoral biomarkers (e.g. NT-proBNP, renal function; see Supplemental *Table 1* for complete list) is directly measured in fresh material, whereas the remaining biosamples are stored semi-automatically in the centralized biobank with electronic temperature monitoring. The biorepository is controlled by sample management software (proprietary development) containing the sample-specific information. Quality documentation is performed for all biomaterials processed and saved electronically in the sample management database. The biomaterial is stored at -80°C in an aliquot system with two-dimensional barcodes. To avoid loss of sample, biomaterials of every participant are stored in two different freezers at two different storage locations with an electronic temperature monitoring and alarm system.

Platelet function analyses

For a subsample of the MyoVasc study, whole blood was taken for analysis of platelet function and platelet phenomics. Fresh citrated whole blood for platelet function analysis was analysed within two hours after blood withdrawal from the follow-up visits of study participants with confirmed HF at their baseline examination. Four different tests of platelet function were carried out, enabling investigations into platelet aggregation, activation and thrombin generation: A platelet function analyser 200 system (Siemens Healthcare, Marburg, Germany) was used to measure platelet aggregation in

Downloaded from https://academic.oup.com/eurjpc/article/28/9/1009/6353474 by guest on 05 September 2024

Table 1 Components of the biannual investigation at the study centre.

	Baseline examination	Two-year follow-up	Four-year follow-up	Six-year follow-up
Computer-assisted personal interview	√	√	√	√
Physical examination	√	√	√	√
Anthropometry	√	√	√	√
Body temperature	√	√	√	√
2D and 3D echocardiography	√	√	√	√
Body plethysmography	√	√	√	√
Vascular function and structure				
Arterial stiffness (digital volume pulse)	√	√	√	√
Digital volume pulse / resistance vessel reactivity	√	√	√	√
Radial artery reactivity (FMD/FMC)	√	√	√	√
Vascular hypertrophy and atherosclerosis				
Ankle-brachial index	√	√	√	√
Sonography of the carotid arteries	√	√	√	√
Cardiopulmonary exercise testing	√	√	√	√
Circulation				
Resting blood pressure and heart rate	√	√	√	√
24-h Holter ambulatory blood pressure measurement	√	√	√	√
Cardiac electrophysiology				
12-lead resting electrocardiogram	√	√	√	√
24-h Holter electrocardiogram	√	√	√	√
Assessment of chronic venous insufficiency	√	√	√	√
Medication (ATC-coded)	√	√	√	√
Meteorological data	√	√	√	√
Blood sampling and biobanking				
Ad hoc lab profile (51 humoral biomarkers)	√	√	√	√
Citrated plasma	√	√	√	√
EDTA plasma	√	√	√	√
Isolation of DNA	√	√	√	√
Isolation of RNA	√	√	√	√
Platelet function testing	√	√	√	√
Serum	√	√	√	√
Urine	√	√	√	√
Questionnaires				
Daily strain	√	√	√	√
Emotional distress (anxiety disorders, depression)	√	√	√	√
Genetic risk	√	√	√	√
Life events	√	√	√	√
Personality	√	√	√	√
Physical activity	√	√	√	√
Quality of life	√	√	√	√
Social integration and phobia	√	√	√	√

Platelet function testing is performed in a subsample of the MyoVasc study only. FMC: flow-mediated constriction of the radial artery; FMD: flow-mediated dilatation of the radial artery.

whole blood at high shear rates after collagen/adenosine diphosphate and collagen/epinephrine stimulation. Platelet aggregation was additionally investigated in undiluted platelet-rich plasma using light transmission aggregometry (LABiTec, Ahrensburg, Germany) after the addition of different platelet agonists (i.e. collagen/adenosine diphosphate, collagen/epinephrine, collagen, arachidonic acid and thrombin receptor activating peptide). A calibrated automated thrombogram assay (Thrombinoscope BV, Maastricht, the Netherlands) was used to measure thrombin generation in platelet-rich plasma (with adjusted platelet concentration to 150,000 platelets/µl using autologous platelet-poor plasma) after exposure to a trigger (e.g. low tissue factor). Activation-dependent platelet surface antigens (i.e. P-selectin,

Downloaded from https://academic.oup.com/eurjpc/article/28/9/1009/6353474 by guest on 05 September 2024

Table 2 Components of the computer-assisted personal and telephone interviews in the MyoVasc study.

	Baseline examination	One-year follow-up	Two-year follow-up	Three-year follow-up	Four-year follow-up	Five-year follow-up	Six-year follow-up
Computer-assisted personal interview							
Medical history – health status and diseases	√		√		√		√
Classical risk factors	√		√		√		√
Disease-related pathology	√		√		√		√
Domestic and family situation							
Family history	√		√		√		√
Gender-specific items	√		√		√		√
Health behaviour	√		√		√		√
Health care utilization	√		√		√		√
Job-related history	√		√		√		√
Life satisfaction and environmental concerns	√		√		√		√
Computer-assisted telephone interview							
Assessment of study outcomes		√	√	√	√	√	√
Cardiovascular risk factors		√	√	√	√	√	√
Clinical status		√	√	√	√	√	√
Comorbidities							
Emotional distress		√	√	√	√	√	√
Heart failure symptoms		√	√	√	√	√	√
Medication		√	√	√	√	√	√

CD63 and Annexin V) at resting conditions in whole blood were analysed by flow cytometry using a BD Accuri C6 system (BD Biosciences, San Jose, USA). In total, 23 platelet characteristics are measured per individual.

Data management and quality control

Central data management was installed, fulfilling the requirements of data protection and data safety. Databases (e.g. person-identifying information, study and laboratory measurements, genetic information and image archive) are electronically and physically separated. Access to databases is centrally controlled, providing individual authorization of staff members for specific databases according to the individual's responsibilities. In order to ensure high-quality data, plausibility checks for completeness and correctness according to predefined procedures and automated integrity checks have been implemented for the assessment of all study obtained in the study centre and via CATI.

Analysis concept

In the conception of the MyoVasc study, a sample size of approximately 2800 subjects was envisaged for the study population, and a sample size of approximately 500 subjects for the control group was intended. The large sample size of the MyoVasc study offers the possibility for the application of different analysis designs (e.g. cross-sectional analysis, longitudinal analysis, hypothesis-based or agnostic approaches) depending on the question under investigation. In general, analysis will be carried out (a) after inclusion of the first 1000 study participants (T1), (b) after the inclusion of 2000 study participants (T2), (c) after recruitment of the total study sample (T3), (d) after completion of the annual follow-up visits by phone (T4), and (e) after completion of the two-year, four-year and six-year follow-up investigation in the study centre for half (T5, T7, T9) and all (T6, T8, T10) eligible study participants.

Power considerations

For the cross-sectional comparison of HF individuals with population controls using a Mann–Whitney *U*-test (two-sided, $\alpha = 0.05$), an effect size of 0.14 for the difference in cardiac function could be detected between groups at 80% power. For investigation of the clinical course of HF, an event rate of 14% for worsening of HF is assumed according to the literature.[17–19] Considering a drop-out rate of 20%, the sample size of the MyoVasc cohort would be sufficient to detect a relative risk of 1.4 comparing exposed and unexposed HF individuals at 80% power given a risk factor prevalence of 20%. Further power considerations related to the accuracy of risk description and the detection of HF risk factors are provided in the Supplemental Appendix.

Systems biomedicine approach to heart failure

Since HF is a multi-factorial syndrome with diverse causes involving complex physiological and pathophysiological processes, a data-driven, systems-medicine approach is ideal to identify groups of individuals with shared pathophysiological mechanisms (see *Figure 3*). The MyoVasc study offers a unique multi-dimensional database with deep clinical and molecular characterization. In addition to the multi-dimensional data obtained within the investigation in the study centre, the first multi-omics molecular data have been generated: (a) the full study sample of the MyoVasc cohort underwent genotyping using commercially available bead chips (i.e. Illumina InfiniumOmniExpressExome-8 v1.3 for the first $n = 2000$ individuals and Illumina Omni EUR HD for the remaining $n = 1289$ individuals;

Downloaded from https://academic.oup.com/eurjpc/article/28/9/1009/6353474 by guest on 05 September 2024

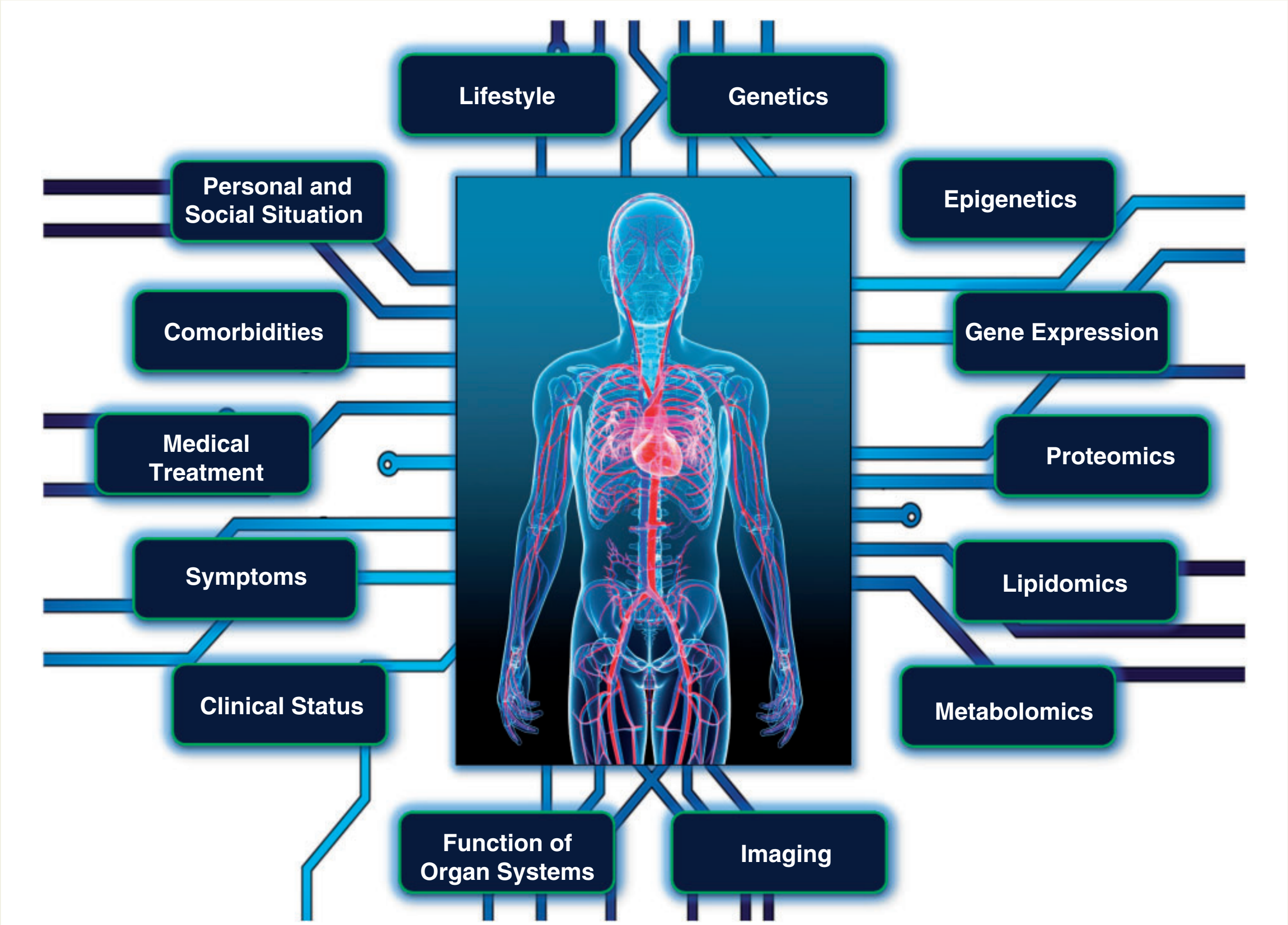

Figure 3 Multi-dimensional characterization of heart failure. Schematic overview of the assessment of study participants in the MyoVasc study by multi-dimensional characterization.

Life & Brain GmbH, Bonn, Germany); (b) in the subsample of the first 1645 HF individuals, RNA-sequencing in whole blood has been performed; (c) high-throughput proteomic profiling of humoral protein biomarkers was conducted in the full cohort via proximity extension assay-based immunoassay panels (Olink®, Uppsala, Sweden). This will allow analysis of phenotypic information, evaluation of molecular data, and identification of signatures by the use of state-of-the-art methods in biostatistics and bioinformatics, including supervised and agnostic unsupervised learning (e.g. machine learning techniques like LASSO regression, random forest and neuronal networks) with subsequent pathway analysis. Appropriate biostatistical methods will be applied to cross-sectional analyses (e.g. logistic and linear regression) as well as prospective investigations evaluating clinical outcome (e.g. multivariable Cox regression).

Baseline characteristics of the MyoVasc study

In total, 3289 subjects were enrolled in the MyoVasc study from January 2013 to April 2018. The baseline characteristics of study participants are presented in *Table 3*. In the study sample, the prevalence of symptomatic HF according to ACC/AHA HF guidelines (i.e. HF stage C/D) was 52.9%, whereas 1253 individuals were at HF stage A/B and 295 subjects were free of HF risk factors. As expected, HF subjects were older, more often male, and had a higher prevalence of cardiovascular risk factors and comorbidities and a higher intake of medication compared with individuals without HF or at risk of HF development. Among individuals with symptomatic HF, HFpEF (37.1%) was the phenotype with the highest prevalence, followed by HFpEF borderline (23.0%) and HFrEF (19.7%). Inhibitors of the renin–angiotensin system (63.5%), antithrombotic agents (61.3%) and beta-blockers (53.7%) were the most frequently recorded medications in the HF study sample.

Discussion

Advances in the management of HF over the past years have led to improved outcomes for HF patients, predominantly in the setting of HFrEF.[20] Although survival after the diagnosis of HF has improved during the past 30 years – reflected by a decline of the age-adjusted death rate19,21 and an increased mean age at death from HF[22] – five-year mortality is still up to 50% and thus worse than that of most malignancies.4,5 Of clinical importance, HF patients present with high

Downloaded from https://academic.oup.com/eurjpc/article/28/9/1009/6353474 by guest on 05 September 2024

Table 3 Baseline characteristics of the MyoVasc study participants.

Sample size, *n*	3289
Demographics	
Age, years	64.6 ± 11.1
Sex (female), % (*n*)	36.8% (1209)
BMI, kg/m^2	28.3 ± 5.0
Waist-to-height ratio	0.58 ± 0.08
Heart failure characteristics	
Systolic blood pressure, mmHg	132.0 ± 18.2
Diastolic blood pressure, mmHg	77.7 ± 10.1
Heart rate, bpm	63.0 ± 10.6
NYHA class	
I, % (*n*)	66.7(2,192)
II, % (*n*)	22.0 (724)
III, % (*n*)	8.6 (283)
IV, % (*n*)	2.6 (85)
HF stages*	
ACC/AHA Stage 0, % (*n*)	9.0 (295)
ACC/AHA HF Stage A, % (*n*)	15.0 (494)
ACC/AHA HF Stage B, % (*n*)	23.1 (759)
ACC/AHA HF Stage C/D, % (*n*)	52.9 (1741)
HFrEF, % (*n*)	19.7 (343)
HFpEF borderline, % (*n*)	23.0 (401)
HFpEF, % (*n*)	37.1 (646)
LVEF, %	54.6 ± 11.0
E/E′ ratio	8.4 (6.4/11.2)
LVMI, g/m$^{2.7}$	44.5 (36.5/54.7)
Medical history	
Atrial fibrillation, % (*n*)	23.0 (756)
Chronic kidney disease, % (*n*)	16.9 (556)
COPD, % (*n*)	12.7 (419)
Coronary artery disease, % (*n*)	37.5 (1235)
PCI, % (*n*)	27.7 (910)
CABG, % (*n*)	10.6 (347)
History of myocardial infarction, % (*n*)	23.7 (781)
Diabetes mellitus, % (*n*)	22.4 (736)
History of TIA/stroke, % (*n*)	8.5 (278)
History of venous thromboembolism, % (*n*)	8.4 (275)
ICD, % (*n*)	8.8 (289)
Pacemaker, % (*n*)	7.3 (240)
Peripheral artery disease, % (*n*)	6.6 (217)
Sleep apnoea, % (*n*)	11.0 (350)
Tumour disease, % (*n*)	16.2 (533)
Medication	
ACE inhibitor/AT1-receptor blocker (C09), % (*n*)	63.5 (2090)
Aldosterone antagonist (C03DA), % (*n*)	13.6 (448)
Antidiabetic medication (A10), % (*n*)	16.4 (540)
Antithrombotic agents (B01), % (*n*)	61.3 (2015)
Beta-blockers (C07), % (*n*)	53.7 (1766)
Cardiac glycosides (C01a), % (*n*)	2.4 (80)
Diuretics (C03), % (*n*)	30.6 (1006)
If channel inhibitor (C01EB17), % (*n*)	5.7 (186)
Lipid-modifying agents (C10), % (*n*)	45.9 (1510)

Continued

Table 3 *Continued*

Sample size, *n*	3289
Humoral biomarkers	
C-reactive protein, mg/l	1.8 (0.9/3.8)
eGFR, ml/min/1.73m^2	78.1 ± 19.5
Haemoglobin, g/dl	14.2 ± 1.4
HbA1c, %	5.7 (5.4/6.1)
NT-proBNP, pg/ml	164.0 (70.0/464.0)
Potassium, mmol/l	4.0 ± 0.4
Sodium, mmol/l	140 ± 2
Troponin I, pg/ml	3.8 (1.9/7.7)

Baseline characteristics of the study sample. Information on clinical characteristics was derived from personal computer-assisted interviews, medical-technical investigations and medical reports. Dependent on distribution, linear trait values are presented either as mean ± standard deviation or as median with interquartile range (25th/75th percentile). Relative and absolute frequencies of categorical variables are presented. Echocardiographic data were assessed during the baseline investigation in the study centre. Systolic function (i.e. LVEF) was assessed using Simpson's method in apical four-chamber view, diastolic function (i.e. E/E′ ratio) was derived from Doppler recordings of mitral inflow (E) and pulsed wave tissue Doppler imaging recordings at the lateral mitral annulus (E′), and left ventricular mass indexed for body height was calculated via a linear method utilizing the cube formula. The 2009 CKD-EPI equation was used to estimate renal function (i.e. eGFR).[40] Information on medication was assessed according to the anatomical therapeutic chemical (ATC) classification system of drugs during the baseline visit in the study centre. ATC codes for the categories of medication are displayed in brackets accompanying the drug's name. The missing rate for all variables is < 1%. *HF phenotypes were classified according to ACC/AHA guidelines.[1] In individuals of ACC/AHA Stage C/D, $n = 351$ were not classifiable to a HF phenotype.

BMI: body mass index; CABG: coronary artery bypass grafting; COPD: chronic obstructive pulmonary disease; eGFR: estimated glomerular filtration rate; HF: heart failure; HFpEF: heart failure with preserved ejection fraction; HFrEF: heart failure with reduced ejection fraction; ICD: implantable cardiac defibrillator; LVEF: left ventricular ejection fraction; LVMI: left ventricular mass index; MI: myocardial infarction; NYHA: New York Heart Association; PCI: percutaneous coronary intervention; TIA: transient ischaemic attack.

inter-individual variability with regard to the development and course of the disease.[20] Consequently, chronic and acute HF continues to impose a tremendous health care burden despite recent treatment advances, especially when considering the estimated increase of disease prevalence due to the ageing of populations.

The relevance of comorbidities for the outcome and management of HF has received increasing attention across the globe.[23] Although multi-morbidity is highly prevalent in HF patients and related to the clinical outcome, previous studies have predominantly focused on the investigation of single, distinct comorbidities in isolation and at a non-granular resolution. This raises a need for comprehensive studies making use of a comprehensive, integrated phenotyping with highly standardized deep clinical and molecular characterization. This is especially relevant for the HFpEF phenotype, which remains the most challenging entity of the HF syndrome for practising clinicians and scientists alike. In the past, several mechanisms have been discussed for the pathophysiology of HFpEF, indicating a complex interplay of extra-cardiac comorbidities (e.g. renal insufficiency, pulmonary disease, systemic inflammation and multi-organ deficiencies) with the cardiovascular system.[24] Especially since currently no specific therapy

Downloaded from https://academic.oup.com/eurjpc/article/28/9/1009/6353474 by guest on 05 September 2024

is available for HFpEF,[25] the lack of a medical therapy for this group represents one of the major unmet clinical needs in the 21st century.[26] However, management of patients with HFrEF also is still challenging and remains unsatisfactory: the persistent high frequency of adverse outcome still indicates a great need for improvements of medical therapy. In this context, several approaches are currently being discussed, aiming to improve adherence to medical therapy and to develop pathway- and cardiac-specific drugs with predictable individual response to therapy.[27–31]

The sample of the MyoVasc study comprises the full range of HF stages. In contrast to other studies and registries in the field, the biodatabase was not restricted to individuals with chronic, symptomatic HF but also included individuals at risk of developing symptomatic HF, enabling the investigation of the transition from asymptomatic to symptomatic HF.28,32,33 Differences between established HF phenotypes are prominent in several dimensions, including the course of the disease and the cause of death.[34] Although sudden cardiac death is an accepted endpoint in many randomized clinical trials on HF, recent studies have demonstrated that differences in death patterns exist between individuals with HFrEF and subjects with HFpEF: HFrEF patients predominantly experience cardiovascular death, whereas HFpEF patients die to a great extent also from various non-cardiovascular conditions, including infection, malignancy, multisystem disease and respiratory disease.[15] Despite heterogeneity in study populations and differences in the definition of HF phenotypes, this clearly indicates a need for advanced phenotypic investigation of individuals, detailed assessment of the clinical course of HF, and identification of risk factors for cardiac and non-cardiac death in specific HF phenotypes.[35]

Due to its heterogeneity and the lack of superior alternative definitions, HF is currently defined as a clinical syndrome rather than a clearly defined disease. Although consensus exists among European and American HF guidelines that subgroups are defined according to the historical echocardiographic measure LVEF, imprecision in the definition of HF remains.1,8 Novel echocardiographic measures (e.g. speckle-tracking echocardiography) and also newer imaging techniques like magnetic-resonance imaging are now available, which might advance diagnostics, management and risk stratification of HF patients.36,37 Advances in high-throughput biomarker measurements and subsequent bioinformatics work-up now enable the conduct of multi-layer phenomapping studies investigating the causes of HF, novel risk factors, the clinical course of the disease and response to therapy.[38] Recently, molecular endophenotypes with variations in clinical HF outcome have been identified within a sample of HFrEF patients by an unsupervised cluster analysis of 92 cardiovascular biomarkers.[39] Future studies are warranted making use of state-of-the-art developments in multi-omics research in order to extend this approach to sequential, high-dimensional comprehensive characterization of HF. The combination of supervised and unbiased approaches offers a unique opportunity to advance current knowledge about HF by moving HF from an imprecise clinical syndrome towards a clearly defined disease with implications for individualized therapy.

Conclusions

Despite major advances in research and improvements in treatment, management of HF patients remains challenging due to a persistently high morbidity and mortality. Current knowledge about the HF syndrome is limited given its large variation in clinical presentation, causation and clinical outcome. Against this background, the high granularity of data from various molecular, subclinical and clinical data levels on individuals with HF in the MyoVasc study will enable researchers to conduct systems-oriented research approaches to close current knowledge gaps in HF research.

Supplementary material

Supplementary material is available at *European Journal of Preventive Cardiology*.

Acknowledgement

We are indebted to the study participants and the team of the study staff of the MyoVasc study and the co-workers of the Clinical Epidemiology of the University Medical Centre Mainz. Part of *Figure 3* is authored by *pixdesign123*.

Declaration of conflicting interests

The authors declared the following potential conflicts of interest with respect to the research, authorship, and/or publication of this article: J.H.P., M.P.N. and P.S.W. report funding for research from the Federal Ministry of Education and Research, Germany (BMBF 01EO1003). P.S.W. received funding from Boehringer Ingelheim, Novartis, Philips Medical Systems, Sanofi-Aventis, Bayer Vital, Daiichi Sankyo Europe, Institute for the Modernization of Economic Base and Employment Structures, Portavita, Federal Institute for Occupational Safety and Health; Health Economy Initiative, Ministry of Health, and Ministry of Economics, Rhineland-Palatinate, Federal Ministry of Health of Rhineland-Palatinate, and the Mainz Heart Foundation; and has also received honoraria for lectures or consulting from Boehringer Ingelheim and Public Health, Heinrich-Heine-University Dusseldorf. The remaining authors declare no conflict of interest.

Funding

The author(s) disclosed receipt of the following financial support for the research, authorship, and/or publication of this article: The MyoVasc study is funded by the German Centre for Cardiovascular Research (DZHK) and the Centre for Translational Vascular Biology (CTVB) of the University Medical Centre Mainz.

References

1. Yancy CWJessup MBozkurt B, *et al.* 2013 ACCF/AHA guideline for the management of heart failure: a report of the American College of Cardiology Foundation/American Heart Association Task Force on practice guidelines. *Circulation* 2013;**128**: e240–327.
2. Stewart SJenkins ABuchan S, *et al.* The current cost of heart failure to the National Health Service in the UK. *Eur J Heart Fail* 2002;**4**: 361–371.
3. MacIntyre KCapewell SStewart S, *et al.* Evidence of improving prognosis in heart failure: Trends in case fatality in 66 547 patients hospitalized between 1986 and 1995. *Circulation* 2000;**102**: 1126–1131.
4. Mosterd ACost BHoes AW, *et al.* The prognosis of heart failure in the general population: The Rotterdam Study. *Eur Heart J* 2001;**22**: 1318–1327.
5. Cowie MRWood DACoats AJ, *et al.*.Survival of patients with a new diagnosis of heart failure: A population based study. *Heart* 2000;**83**: 505–510.
6. Stewart SMacIntyre KHole DJ, *et al.* More 'malignant' than cancer? Five-year survival following a first admission for heart failure. *Eur J Heart Fail* 2001;**3**: 315–322.

Downloaded from https://academic.oup.com/eurjpc/article/28/9/1009/6353474 by guest on 05 September 2024

7. Hobbs FDKenkre JERoalfe AK, *et al.* Impact of heart failure and left ventricular systolic dysfunction on quality of life: A cross-sectional study comparing common chronic cardiac and medical disorders and a representative adult population. *Eur Heart J* 2002;**23**: 1867–1876.
8. Ponikowski PVoors AAAnker SD, *et al.* 2016 ESC Guidelines for the diagnosis and treatment of acute and chronic heart failure: The Task Force for the diagnosis and treatment of acute and chronic heart failure of the European Society of Cardiology (ESC). Developed with the special contribution of the Heart Failure Association (HFA) of the ESC. *Eur Heart J* 2016;**37**: 2129–2200.
9. Gheorghiade MAbraham WTAlbert NM, *et al.* Systolic blood pressure at admission, clinical characteristics, and outcomes in patients hospitalized with acute heart failure. *JAMA* 2006;**296**: 2217–2226.
10. Nave AHLange KSLeonards CO, *et al.* Lipoprotein (a) as a risk factor for ischemic stroke: A meta-analysis. *Atherosclerosis* 2015;**242**: 496–503.
11. Redfield MMJacobsen SJBurnett JCJr., *et al.* Burden of systolic and diastolic ventricular dysfunction in the community: Appreciating the scope of the heart failure epidemic. *JAMA* 2003;**289**: 194–202.
12. Paulus WJTschope CSanderson JE, *et al.* How to diagnose diastolic heart failure: A consensus statement on the diagnosis of heart failure with normal left ventricular ejection fraction by the Heart Failure and Echocardiography Associations of the European Society of Cardiology. *Eur Heart J* 2007;**28**: 2539–2550.
13. Bursi FWeston SARedfield MM, *et al.* Systolic and diastolic heart failure in the community. *JAMA* 2006;**296**: 2209–2216.
14. Bhatia RSTu JVLee DS, *et al.* Outcome of heart failure with preserved ejection fraction in a population-based study. *N Engl J Med* 2006;**355**: 260–269.
15. Vaduganathan MPatel RBMichel A, *et al.* Mode of death in heart failure with preserved ejection fraction. *J Am Coll Cardiol* 2017;**69**: 556–569.
16. Redfield MM.Heart failure with preserved ejection fraction. *N Engl J Med* 2016; **375**: 1868–1877.
17. Maggioni APDahlstrom UFilippatos G, *et al.* EURObservational Research Programme: Regional differences and 1-year follow-up results of the Heart Failure Pilot Survey (ESC-HF Pilot). *Eur J Heart Fail* 2013;**15**: 808–817.
18. Crespo-Leiro MGAnker SDMaggioni AP, *et al.* European Society of Cardiology Heart Failure Long-Term Registry (ESC-HF-LT): 1-year follow-up outcomes and differences across regions. *Eur J Heart Fail* 2016;**18**: 613–625.
19. Jhund PSMacintyre KSimpson CR, *et al.* Long-term trends in first hospitalization for heart failure and subsequent survival between 1986 and 2003: A population study of 5.1 million people. *Circulation* 2009;**119**: 515–523.
20. Metra MTeerlink JR.Heart failure. *Lancet* 2017;**390**: 1981–1995.
21. Levy DKenchaiah SLarson MG, *et al.* Long-term trends in the incidence of and survival with heart failure. *N Engl J Med* 2002;**347**: 1397–1402.
22. Laribi SAouba ANikolaou M, *et al.* Trends in death attributed to heart failure over the past two decades in Europe. *Eur J Heart Fail* 2012;**14**: 234–239.
23. Tromp JTay WTOuwerkerk W, *et al.* Multimorbidity in patients with heart failure from 11 Asian regions: A prospective cohort study using the ASIAN-HF registry. *PLoS Med* 2018;**15**: e1002541.
24. Sharma KKass DA.Heart failure with preserved ejection fraction: Mechanisms, clinical features, and therapies. *Circ Res* 2014;**115**: 79–96.
25. Butler JFonarow GCZile MR, *et al.* Developing therapies for heart failure with preserved ejection fraction: Current state and future directions. *JACC Heart Fail* 2014;**2**: 97–112.
26. Shah AMMann DL.In search of new therapeutic targets and strategies for heart failure: Recent advances in basic science. *Lancet* 2011;**378**: 704–712.
27. McMurray JJ.CONSENSUS to EMPHASIS: The overwhelming evidence which makes blockade of the renin-angiotensin-aldosterone system the cornerstone of therapy for systolic heart failure. *Eur J Heart Fail* 2011;**13**: 929–936.
28. Voors AAAnker SDCleland JG, *et al.* A systems BIOlogy Study to TAilored Treatment in Chronic Heart Failure: Rationale, design, and baseline characteristics of BIOSTAT-CHF. *Eur J Heart Fail* 2016;**18**: 716–726.
29. Tromp JWestenbrink BDOuwerkerk W, *et al.* Identifying pathophysiological mechanisms in heart failure with reduced versus preserved ejection fraction. *J Am Coll Cardiol* 2018;**72**: 1081–1090.
30. Andersen JGerds TAGislason G, *et al.* Socioeconomic position and one-year mortality risk among patients with heart failure: A nationwide register-based cohort study. *Eur J Prev Cardiol* 2020;**27**: 79–88.
31. Scalvini SGrossetti FPaganoni AM, *et al.* Impact of in-hospital cardiac rehabilitation on mortality and readmissions in heart failure: A population study in Lombardy, Italy, from 2005 to 2012. *Eur J Prev Cardiol* 2019;**26**: 808–817.
32. Bossone EArcopinto MIacoviello M, *et al.* Multiple hormonal and metabolic deficiency syndrome in chronic heart failure: Rationale, design, and demographic characteristics of the T.O.S.CA. Registry. *Intern Emerg Med* 2018;**13**: 661–671.
33. van Boven NAkkerhuis KMAnroedh SS, *et al.* Serially measured circulating miR-22-3p is a biomarker for adverse clinical outcome in patients with chronic heart failure: The Bio-SHiFT study. *Int J Cardiol* 2017;**235**: 124–132.
34. Lam CSDonal EKraigher-Krainer E, *et al.* Epidemiology and clinical course of heart failure with preserved ejection fraction. *Eur J Heart Fail* 2011;**13**: 18–28.
35. Lam CSAnand IZhang S, *et al.* Asian Sudden Cardiac Death in Heart Failure (ASIAN-HF) registry. *Eur J Heart Fail* 2013;**15**: 928–936.
36. Park JJPark JBPark JH, *et al.* Global longitudinal strain to predict mortality in patients with acute heart failure. *J Am Coll Cardiol* 2018;**71**: 1947–1957.
37. Shah AMClaggett BSweitzer NK, *et al.* Prognostic importance of impaired systolic function in heart failure with preserved ejection fraction and the impact of spironolactone. *Circulation* 2015;**132**: 402–414.
38. Ferreira JPDuarte KMcMurray JJV, *et al.* Data-driven approach to identify subgroups of heart failure with reduced ejection fraction patients with different prognoses and aldosterone antagonist response patterns. *Circ Heart Fail* 2018;**11**: e004926.
39. Tromp JOuwerkerk WDemissei BG, *et al.* Novel endotypes in heart failure: Effects on guideline-directed medical therapy. *Eur Heart J* 2018;**39**: 4269–4276.
40. Levey ASStevens LASchmid CH, *et al.* A new equation to estimate glomerular filtration rate. *Ann Intern Med* 2009;**150**: 604–612.

Downloaded from https://academic.oup.com/eurjpc/article/28/9/1009/6353474 by guest on 05 September 2024
